# Biocomposite Cryogels for Photothermal Decontamination of Water

**DOI:** 10.1021/acs.langmuir.3c00623

**Published:** 2023-05-26

**Authors:** Muhammad S. Zafar, Francesca Gatto, Giorgio Mancini, Simone Lauciello, Pier P. Pompa, Athanassia Athanassiou, Despina Fragouli

**Affiliations:** † Smart Materials, Istituto Italiano di Tecnologia, via Morego 30, 16163 Genova, Italy; ‡ Dipartimento di Informatica, Bioingegneria, Robotica e Ingegneria dei Sistemi (DIBRIS), Università degli Studi di Genova, Via Opera Pia 13, 16145 Genova, Italy; § Nanobiointeractions & Nanodiagnostics, Istituto Italiano di Tecnologia, via Morego 30, 16163 Genova, Italy; ∥ Electron Microscopy Facility, Istituto Italiano di Tecnologia, via Morego 30, 16163 Genova, Italy

## Abstract

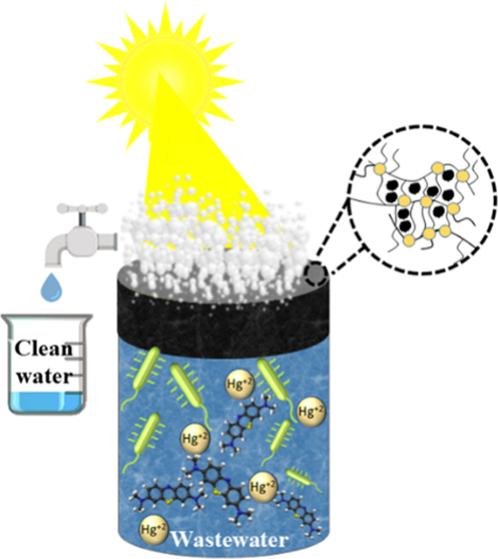

An effective and sustainable approach to deal with the scarcity of freshwater is interfacial solar-driven evaporation. Nonetheless, some serious challenges for photothermal materials still need to be considered, such as long-term stability in harsh environments, eco-friendly materials, and cost-effective and simple fabrication processes. Keeping these points in mind, we present a multifunctional silver-coated vegetable waste biocomposite cryogel that not only exhibits high porosity and enhanced wettability and stability but also possesses high light absorption and low thermal conductivity favorable for heat localization, solar steam generation, and efficient photothermal conversion efficiency. The achieved solar evaporation rate is 1.17 kg m^–2^ h^–1^ with a solar-to-vapor conversion efficiency of 81.11% under 1 Sun irradiation. The developed material is able to effectively desalinate artificial seawater and decontaminate synthetic wastewater (e.g., water containing dye molecules and mercury ions) with an efficiency of >99%. Most importantly, the composite cryogel presents antifouling properties, and in particular, salt antifouling ability and anti-biofouling properties. Thus, the numerous functionalities of the biocomposite cryogel make it a cost-effective promising device for prolonged water decontamination processes.

## Introduction

The limited pure water availability and the depletion of its resources affect almost 3 billion people.^1−3^ For this reason, research is focused on finding new strategies for the recovery of clean water from unconventional resources, such as seawater or wastewater, via low-cost, efficient, and reliable strategies. Specifically, water purification through evaporation using solar light as the main energy source has great potential to become a sustainable solution to water scarcity.^4,5^ Nonetheless, conventional water evaporation approaches have important drawbacks that markedly limit their efficient application. In particular, due to the fact that the heating process takes place in the entire water body, conventional water evaporation approaches present low solar-vapor conversion efficiency.^6,7^ To deal with this issue, interfacial solar evaporation processes have been developed^7^ using materials able to locally heat the water/air interface for a rapid and efficient performance with minimal heat losses. To this aim, highly efficient solar steam generators have been developed through careful material design and development for efficient light absorption, appropriate wettability, and improved thermal management.^8^

Until now, several photothermal materials for effective solar steam generation and desalination have been developed, such as semiconductors,^9−12^ plasmonic,^13−15^ and carbon-based materials,^16−25^ combined, or not, with solid porous supports of natural or synthetic nature. Although many of them present high performances, they involve expensive materials and, in some cases, hazardous chemicals, and complex and energy-consuming fabrication processes,^9,15,26−29^ limiting their large-scale production and compromising the sustainable concept of the solar-driven photothermal action. Alternatively, hydrophilic polymers (e.g., poly(vinyl alcohol), polysaccharides, poly(acrylic acid), and so on) in the form of three-dimensional (3D) porous foams, hydrogels, or cryogels, combined with low-cost components able to absorb and transform solar light into heat, depict valuable platforms for the fabrication of interfacial solar evaporation components.^30−36^

One of the components that can effectively transform solar light into heat is biochar, the carbon-based material prepared by the thermal treatment of biomass.^19,20,37,38^ Because of the variety in raw biomass, several polymeric, biochar-based functional composites have been developed, showing economical, scalable, and eco-friendly characteristics for applications in water treatment, energy storage, and catalysis.^1,39−42^

One widely used vegetable is beetroot (*Beta vulgaris*), with an annual production of more than 200,000 tons only in Western Europe. Almost 90% of it is utilized as food, while the rest is used as a food colorant and for the production of juices.^43^ After processing, a large amount of beetroot waste is produced in the form of pomace, flesh, crown, and peels,^44^ which can be utilized as valuable biomass for the fabrication of advanced materials.^45^

In this work, biochar from beetroot has been used for the development of composite cryogels coated with antibacterial silver nanoparticles (AgNPs), and their performance as interfacial solar steam generators has been explored. The dependence of the cryogels’ performance on their composition has been studied following several characterization techniques. The cryogels were proved to be mechanically and thermally stable, with low thermal conductivity in the dry state, enhanced wettability, and a distinctive porous structure, characteristics which make them promising candidates for water harvesting. Most importantly, they present effective salt rejection upon water desalination, high efficiency to purify water from mercury ions and dye molecules, and good performance stability, demonstrating their potentiality in photothermal purification applications. In addition, the developed materials have antifouling properties, as salt and bacteria do not significantly affect their performance. The herein-presented cost-effective, stable, self-desalting, and antibacterial biochar-based cryogels open up new possibilities for the utilization of any biowaste in the fabrication of valuable components for water harvesting applications.

## Experimental Section

### Materials

Poly(vinyl alcohol) (PVA, *M*_w_ 89,000–98,000 g mol^–1^, 99% hydrolyzed), poly(vinylpyrrolidone) (PVP, *M*_w_ 40,000 g mol^–1^), methylene blue (MB, *M*_w_ 319.85 g mol^–1^), silver nitrate (AgNO_3_, *M*_w_ 169.87 g mol^–1^, 99.0%), mercury(II) chloride (*M*_w_ 271.50 g mol^–1^, 99.55%), mineral oil (μ: 41–17 cSt), silicone oil (μ: 500 cSt), and sea salt (NutriSelect Basic, S9883) were purchased from Sigma-Aldrich and were used without any further purification. Soybean oil (Salvadori, μ: 43 cSt) was purchased from a local market. MilliQ water was used in all experiments. Methyl orange (MO, *M*_w_: 327.33 g mol^–1^) was purchased from Fluka Analytical. Beetroot waste was provided from a local fruit and vegetable market in Genoa, Italy.

### Biochar Preparation

Thin slices of beetroot waste were lyophilized for 48 h (i.e., 0.6 mbar at 10 °C for 40 h and 0.1 mbar at 20 °C for 8 h) (CHRIST Epsilon 2-4 LSCplus, Germany). Subsequently, the samples were carbonized in an atmospheric furnace (Nabertherm GmbH L 9/11/B 410, Germany) at 300 °C for 30 min (heating rate: 5 °C min^–1^). Finally, the carbonized beetroot slices were ground into a fine powder using a mortar and pestle. The carbonized beetroot powder (CB) has particles with a mean size of 33.89 ± 10.20 μm, and its morphology and particle size distribution are shown in Figure S1 of the Supporting Information.

### Fabrication of the Cryogels

PVA (1.0 g) and PVP (0.2 g) (weight ratio 5:1) were added into 10 mL of MilliQ water and dissolved with the help of a microwave oven (Panasonic NN-K101WM, operating power: 240 W) for a few seconds. Subsequently, the gel solution was poured into a Petri dish and mixed manually with 5 g of CB powder. The mixture was placed in a freezer overnight and subsequently at ambient conditions for 3 h to facilitate the cross-linking between PVA and PVP. Then, the as-prepared gel (Supporting Information, Figure S2a) was dipped in MilliQ water at 50 °C and left for 7 h (continuously changing the water every 1 h) to remove the CB traces and impurities. The samples were then freeze-dried (Base Unit LIO5P4K, Italy) at −50 °C and 0.5–0.3 mbar for 48 h to form the cryogel (Supporting Information, Figure S2b). Following the same process but without adding a filler, a PVA/PVP cryogel (BC0) was also formed for comparison reasons. The biocomposite cryogel was named BC5 in accordance with the amount of CB powder (5 g) introduced in the PVA/PVP solution (final concentration of the CB in BC5: 80.6 wt %). It should be mentioned that the choice of this composition is done through preliminary studies on samples with different amounts of CB in the composites (3, 5, and 7 g (BC3, BC5, BC7, respectively)) that revealed that a higher amount of the CB (BC7) compromises the porosity of the final structure, while lower amounts (BC3) negatively affect the photothermal performance (data not shown).

### Decoration of the Cryogel with Silver Nanoparticles

The decoration of the cryogels with AgNPs succeeded following the process reported in ref (46) after slight modifications. In particular, the BC5 cryogels (diameter: 18–19 mm; thickness: 2.5 mm; weight: 0.15 g) were dipped into 30 mL of AgNO_3_ aqueous solutions of different concentrations (0.01, 0.05, 0.10, and 0.20 M) for 24 h. Then, the samples were placed in an oven for 30 min at 110 °C and were left to naturally cool down. The samples were named according to the molar concentrations of the Ag dipping solutions as BC5AgNPs_0.01M_, BC5AgNPs_0.05M_, BC5AgNPs_0.1M_, and BC5AgNPs_0.2M_, and the overall experimental process for their fabrication is presented in Supporting Information Figure S2c.

### Material Characterization

The samples were studied by field emission scanning electron microscopy (FE-SEM) using a JEOL JSM-7500 FA (Jeol, Tokyo, Japan) operating at a 10 kV acceleration voltage and considering back-scattering electrons for better visualizing the chemical composition differences. Before analyses, a sputter coater (EMITECH K950X) was used to coat the samples with a 10 nm carbon layer. Energy-dispersive spectroscopy (EDS, Oxford instrument, X-Max, 80 mm^2^) was used to distinguish the presence of elements in the samples. The morphology of cryogels was also studied by SEM (JEOL JSM-6490LA) operating at 10 kV of acceleration voltage and using secondary electrons. Before analyses, a sputter coater (Cressington 208 HR) was used to apply a 10 nm Au layer on the samples. Pore size distribution, the mean pore size, and the mean AgNPs size were defined by analyzing 90 pores from the SEM images using the ImageJ software.

To explore their chemical properties, the samples were analyzed using an attenuated total reflection (ATR) accessory (MIRacle ATR, PIKE Technologies) coupled to a Fourier transform infrared (FTIR) spectrometer (Vertex 70v FTIR, Bruker). The presented spectra were the average of 128 repetitive scans recorded in the range from 4000 to 600 cm^–1^ with a resolution of 4 cm^–1^. The chemical bonds were characterized using X-ray photoelectron spectroscopy (XPS) with a Thermo Scientific ESCALAB 250Xi X spectrometer, using monochromatic Al Kα (1486.6 eV) X-ray as the light source. Raman measurements were carried out using a HORIBA (HR800 UV) spectrometer with a ×50 objective (numerical aperture of 0.75), an excitation wavelength of 514 nm line of an Ar^+^ laser, and an incident power less than 1 mW. X-ray diffraction (XRD) analyses were conducted using a Malvern PANalytical Empyrean, equipped with a 1.8 kW Cu Kα ceramic X-ray tube and a PIXcel^3D^ 2 × 2 area detector, operating at 45 kV and 40 mA. For the measurement, samples were placed on a zero-diffraction silica wafer.

The photothermal properties of the cryogels during light irradiation were explored using an infrared camera (IR camera, FLIR X6580 sc) and, afterward, analyzed by ResearchIR software. Through-plane thermal conductivity of the BCs was investigated by a modified transient-plane source technique on a thermal conductivity analyzer (C-Therm Technologies, TCi) following the ASTM D7984. The thermal stability of the samples was explored by thermogravimetric analyses (TGA) using a Q500 analyzer (TA Instruments) with a heating rate of 10 °C min^–1^, from 30 to 800 °C in a N_2_ atmosphere.

The skeletal density of the BCs was determined using a pycnometer (Pycnomatic ATC). To do so, parts of each sample (∼0.5 g) were placed in a 4 cm^3^ cuvette and suspended in the pycnometer at 20.00 ± 0.01 °C, using helium as a measuring gas. The measurements were repeated 10 times for each sample, and the accuracy was set to be ±0.5%. The obtained density was then used to calculate the effective porosity and pore size distribution using mercury intrusion porosimetry (MIP, Thermo Fisher Scientific). Measurements were conducted using both low-pressure (Pascal 140 Evo, pressure range: 0.001–0.400 MPa) and high-pressure (Pascal 240 Evo, pressure range: 0.1–200 MPa) modules with a standard dilatometer. The data obtained from both modules were combined and correlated to an equivalent pore size range of 0.01–100.00 μm using S.OL.I.D Evo software. The pore size was calculated using the Washburn equation assuming a cylindrical and plate model, a mercury contact angle, and mercury surface tension of 140° and 0.48 N m^–1^, respectively.

The absorption spectra of samples were recorded using a UV–VIS–NIR spectrophotometer (Varian Cary 5000) equipped with an integrating sphere as the background over a range of 250–2500 nm. The optical absorption efficiency (*A*) was measured according to eq 1
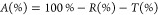
1where *R* and *T* are the percentages of the reflectance and transmittance of the sample, respectively. The percentages of the reflectance and transmittance were determined via integrating the area under the spectra.

### Water Absorption Capacity and Oleophobicity under Water

To remove the adsorbed moisture from the cryogels, a vacuum desiccator was used. After that, the samples were immersed in 10 mL of MilliQ water for defined time intervals (e.g., 0.08, 0.25, 0.5, 1, 3, 5, and 15 h) at ambient conditions. Subsequently, the cryogels were placed gently onto the wet tissue to remove the excess water from the surfaces. The weight of each sample was monitored using a weighing balance in order to calculate the water absorption capacity (*Q*) following eq 2.
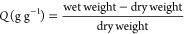
2For each cryogel type, the *Q* was presented as the average value of three different samples.

The wetting properties were studied by measuring the water contact angle (drop volume: 5 μL) and the underwater oil contact angle using a Dataphysics OCA 20 contact angle goniometer at ambient conditions. For the underwater oil contact angle, a square glass chamber was filled with 6.5 mL of MilliQ water, and the sample was immersed slightly below the surface of the water after being fastened on a glass strip. Then, 6–20 μL of oil droplets were dispensed inside the water using an upward needle, which was deposited on the surface of the sample. Underwater roll-off angles were determined by tilting the automatic stage at a rate of 1.5° s^–1^. Each value presented is the mean value of five measurements at different areas of the same sample.

### Photoinduced Steam Generation and Desalination

The experiments were typically conducted at an ambience of 16 ± 1 °C and relative humidity of 36 ± 1%. The dry samples were allowed to float on water in a glass beaker and illuminated under a solar simulator (ScienceTech SLB-150B (Class BAA) with an AM1.5G air mass filter and calibrated by an Oriel reference solar cell and meter (91 150 V)). The mass loss of water was measured using an analytical balance Sartorius (ENTRIS623I-1S, *d* = 0.001 g and Max = 620 g, Lab Instruments GmbH & Co). The solar evaporation rate (SER) was measured by the slope of the water mass loss vs time under 1 Sun (1 kW m^–2^) irradiation for 60 min, while the absolute SER (*ṁ*) was obtained by deducting the bulk water evaporation rate in the dark from the measured rates. As a control, SER of only water was determined as well, under 1 Sun irradiation.

The solar-to-vapor conversion efficiency (η) was calculated according to eq 3(47)
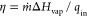
3Here, *ṁ* is the absolute evaporation rate (kg m^–2^ h^–1^), Δ*H*_vap_ is the evaporation enthalpy of the liquid–vapor phase change (eq 4), and *q*_in_ is the power of solar irradiation (1 kW m^–2^).
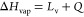
4*L*_v_ is the latent heat of water evaporation, and *Q* is the sensible heat of water. *L*_v_ depends on the temperature of the air/water interface at which water evaporation occurs and ranges from 2256.4 kJ kg^–1^ at 100 °C to 2453.5 kJ kg^–1^ at 20 °C, while *Q* is calculated by multiplying the specific heat capacity of water (4.18 kJ kg^–1^ °C^–1^) with the temperature difference (Δ*T* in °C) of the bulk water and the air/water interface.

Synthetic seawater was prepared by adding sea salt to MilliQ water (3.5 and 10 wt %). The desalination test was conducted in a glass-made condensation chamber. The concentrations of primary ions such as Na^+^, K^+^, Ca^2+^, Mg^2+^, and Sr^+^ in the synthetic seawater and the collected distillate were examined by an inductively coupled plasma optical emission spectrometer (ICP-OES) (iCAP 6300, Thermo Scientific).

### Solar Decontamination of Wastewater

Thanks to their self-floating ability, the samples were floating for 7 h under 1 Sun irradiation on the water containing mercury salt (10 mg L^–1^) or/and MB (30 mg L^–1^) and MO (30 mg L^–1^). The concentration of Hg^+2^ in the collected distillate was measured by ICP-OES, while the concentration of the dyes was evaluated by a UV–vis–NIR spectrophotometer (Varian Cary 6000i) after correlating the concentration of the dyes with the absorption intensity of the characteristic peaks of the dyes (further details in Section S1, of the Supporting Information).

### Self-Desalting Performance of BCs

The salt fouling on the cryogels was explored by simulating an alternation of “day” and “night” during long-term evaporation of water from brine solutions (3.5 and 10 wt %). The evaporation was monitored every 30 min for 7 h under 1 Sun irradiation (to mimic “day”), while irradiation was switched off for 17 h, resting at “night” time. The same procedure was repeated for 3 days (i.e., 72 h). The surface of the samples was monitored through optical microscopy (LEICA DM 2500M). The SER was also measured at specific time points.

### Antibacterial Performance

The antibacterial capability of the BC5 and Ag-coated BC5 samples was evaluated using two different bacterial species: *Escherichia coli* as the representative strain of Gram-negative bacteria and *Enterococcus faecalis* as the representative strain of Gram-positive bacteria. Samples were cut into small pieces (diameter: 1 cm^2^ and weight: 0.065 g) and submerged in a starting bacterial suspension (1 × 10^5^ CFU (colony forming unit) mL^–1^) in LB (Luria–Bertani) medium. The bacterial growth in the presence or absence of the different samples was evaluated through optical density (O.D.) measurements at 600 nm (OD_600_). The suspensions were incubated for up to 5 h at 37 °C under continuous shaking (200 rpm), and the O.D. was taken every hour. After the liquid bacterial culture, the antibacterial behavior was also confirmed using a solid culture medium. Briefly, 100 μL aliquots of the resulting bacterial suspensions after 5 h contact with the samples were uniformly spread onto LB-agar plates. After overnight incubation at 37 °C in a static incubator, the bacterial colonies’ growth on plates was evaluated through visual inspection.

### Biofouling Properties

For the definition of the biofouling properties, the BC5 and AgNP-coated BC5 cryogels were positioned in a glass chamber, floating on a bacterial suspension (*E. coli* 1 × 10^5^ CFU mL^–1^ in LB medium) under 1 Sun irradiation for 1.5 h. During the solar exposure also, the SER was monitored. After their removal from the solution, the samples were incubated overnight in an LB medium at 37 °C. Subsequently, the bacterial growth was evaluated through OD_600_ measurements, and to visually determine the bacterial presence on the samples, SEM analysis was performed. Briefly, samples were fixed with 1.5% glutaraldehyde in 0.1 M sodium-cacodylate buffer for 2 h at room temperature and post-fixed in 1% osmium tetroxide in MilliQ water. After several washes in MilliQ water, the samples were dehydrated in increasing ethanol solutions (starting from 30 to 100%), 1:1 ethanol/hexamethyldisilazane (HDMS, Sigma-Aldrich) solutions, and 100% HMDS and air-dried. Samples were finally coated with a 10 nm thick film of gold and analyzed using a JEOL JSM-6490LA SEM with an acceleration voltage of 10 kV.

## Results and Discussion

**Figure 1:**
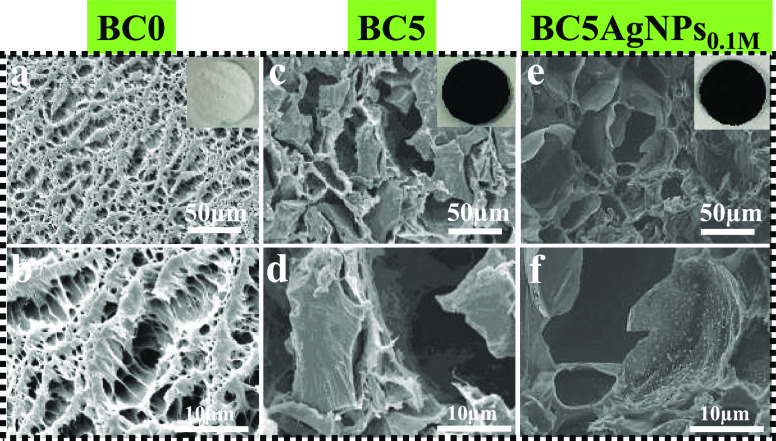
Cross-sectional SEM images of (a, b) BC0, (c, d) BC5, and (e, f) BC5AgNPs_0.1M_. Insets of (a), (c), and (e): digital photographs of the BC0, BC5, and BC5AgNPs_0.1M_ (diameter: 90.89 mm; thickness: 2.5 mm).

As shown in the cross-sectional SEM images of Figure 1, all samples exhibit a highly porous morphology with macropores and interlinked macro channels. In the composites cryogels, the CB filler appears well entrapped within the polymer network, and it clearly affects the overall pore structure of the cryogels. In particular, compared to BC0, the pores of BC5 seem bigger and irregular in size and morphology, while the pore walls are thicker (Figure 1a–d). The top surface of the samples also presents a highly porous structure, and the incorporation of CB filler results in the formation of composite cryogels with bigger pore sizes and a significantly different morphology compared to the bare polymer sample. (Supporting Information Figure S3). In fact, in such cases, the amount of the CB powder is by far greater than the polymer component (80.6 wt % with respect to the composite), and therefore, it is expected that the polymer’s role is to keep together the CB micrometric powder in the form of a robust porous composite. This is also proved by the analysis of the mechanical properties of BC5, which exhibits a compressive modulus of ca. 6.9 MPa and a compressive stress of 14.9 MPa (Figure S4 and Table S1).

To investigate the porosity and pore size distribution of the cryogels in the macro range, MIP analysis was performed. As shown in the intrusion/extrusion isotherms of Figure S5a,b, a large amount of mercury is entrapped into the samples, confirming the highly interconnected porous morphology. The porosity of BC0 and BC5 is 80.89 and 74.64%, respectively. Although the pore size distribution lies between 0.1–100.00 and 0.01–100.00 μm for the BC0 and BC5, respectively, the maximum contribution toward porosity depends on pores having diameters from 0.8 to 10.0 μm for both samples (Figure S5c,d). Therefore, the addition of the CB filler affects the pore size distribution range, whereas it slightly reduces the overall porosity of BC5.

To explore the type of the cryogels’ carbon-based filler (biochar), Raman spectroscopy was performed. As shown in Figure S6, CB and BC5 display two intense peaks representative of the carbon–carbon vibrations observed at 1333/1352 cm^–1^ for the CB and BC5, respectively, and at 1578 cm^–1^ for both samples. The first peak is attributed to the amorphous carbon (D), which is characteristic of sp^3^ defects, whereas the characteristic peak of the graphite structure (G) at 1578 cm^–1^ is attributed to the sp^2^ bonded graphitic carbon.^48^

To induce the antibacterial activity to the cryogel, the BC5 was subsequently dipped in a Ag precursor solution at different concentrations, and after the thermal treatment, as described in the Experimental Section, the material was explored by SEM in order to confirm the presence of the AgNPs. As shown in the representative analysis of Figure 1e–f, for BC5AgNPs_0.1M_, AgNPs of size ranging between a few tens and a few hundreds of nm (average particle size of 250 ± 180 nm) (Supporting Information Figure S7) were successfully formed through BC5 (Figure S3), while the porous structure of the cryogels is well maintained. In fact, according to Sau and Kundu,^46^ PVA acts both as a reducing and a stabilizing agent for the synthesis of AgNPs upon heat treatment with the nanocomposite structure to remain stable without any significant modification in the structure and properties of the pure polymer. As shown in the SEM and EDS analyses of Figures S7 and S8, Supporting Information, the formed AgNPs, after dipping the BC5 in solutions of different concentrations of Ag ions, do not present significant differences in the size and surface density of the formed NPs.

To further study the AgNPs formed on the BC5 sample, XRD analysis was performed (Figure S9). The cryogel samples show an intense broad peak between 16 and 30° (centered at 19.70°), which can be attributed to the (002) diffraction face, indicating the presence of carbon of amorphous nature with a low graphitization degree. Another broad peak is observed between 35 and 45° (centered at 40.49°), indicating the presence of a highly disordered material in the form of carbon. In particular, such a peak can be attributed to the (10) bidimensional plane due to the turbostratic stacking of the hexagonal layers of the carbon atoms, resulting in a disordered structure and low crystallinity.^49,50^ After coating the BC5 with the AgNPs, sharp peaks at 2θ of 33.27, 38.05, 44.29, and 64.44° appear, attributed to the Ag cubic structured crystallographic planes of 122, 111, 200, and 220, respectively.^51,52^ Thus, the XRD pattern demonstrates that the formed AgNPs are crystalline in nature, with a crystallite size of 10.48, 11.75, 8.85, and 10.65 nm for BC5AgNPs_0.01M_, BC5AgNPs_0.05M_, BC5AgNPs_0.1M_, BC5AgNPs_0.2M_, respectively, as defined by the Debye–Scherrer equation (Supporting Information Figure S9 and related discussion).

**Figure 2:**
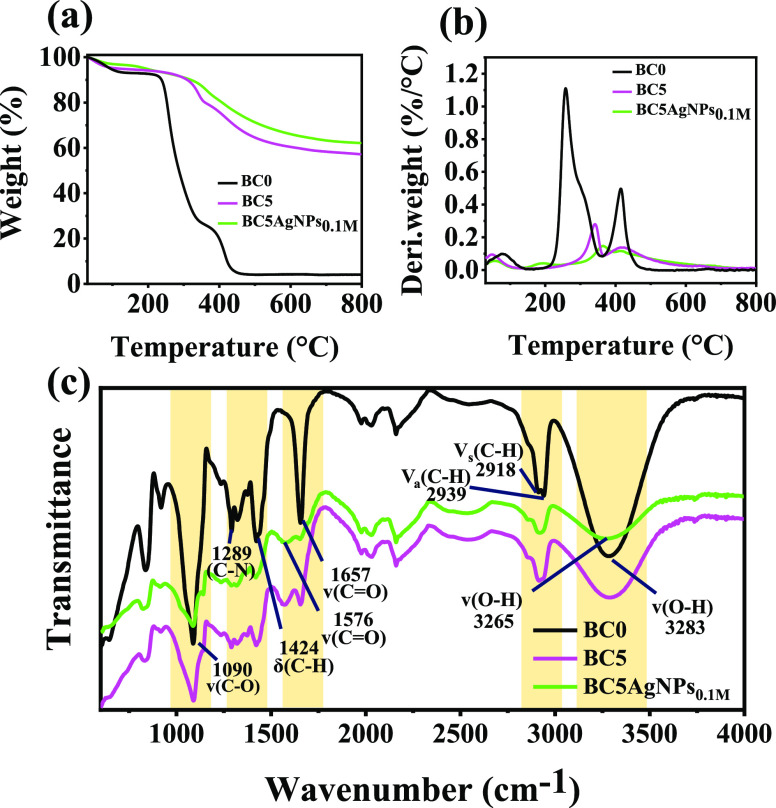
(a) TGA, (b) DTGA, and (c) FTIR spectra of the BC samples.

The thermal stability of the BC5 and BC5AgNP samples was defined by TGA, as shown in Figure 2a, where also the thermal behavior of the BC0 is presented. In all cases, the first weight loss step, up to 100 °C, is attributed to the adsorbed moisture desorption. The second decomposition step of the BC0 is attributed to the chain-striping removal of water from the PVA structure leading to polyene,^53^ (*T*_max_: 260 °C, with a final weight loss of 70.2%), and the third one at *T*_max_: 415 °C (with maximum weight loss of 21.3%), to the breakdown of the carbon structure of the PVA and PVP chains.^54,55^ When the CB is introduced in the polymer structure (i.e., sample BC5), the *T*_max_ representative of the PVA structure decomposition is significantly displaced to higher temperatures (*T*_max_: 341 °C), while the second one, attributed to the PVA and PVP chains’ carbon structure breakdown, is observed at *T*_max_: 420 °C, indicating that the presence of the CB enhances the thermal stability of the polymer structure (Figure 2b). It should be mentioned that the thermal degradation of the pure CB filler also presents a degradation step at 424 °C attributed to the organic mass decomposition remaining in the CB after the carbonization process (Supporting Information Figure S10a and Table S2). In the presence of AgNPs in the BC5, the first main degradation step of the polymer matrix is further displaced toward higher temperatures indicating that the AgNPs further improve the thermal stability of the composite (*T*_max_: 365 °C for the BC5AgNPs_0.1M_). However, an additional degradation step with a *T*_max_ value at 190 °C is observed, possibly attributed to the nonreacted Ag precursor. In fact, as shown in the Supporting Information Figure S10a, the higher the concentration of the precursor solution for the BC5 treatment in order to grow AgNPs, the more evident the presence of this step compared to the overall behavior of the samples. This is in accordance with the XPS analysis (Supporting Information Figure S11 and Table S3), which shows that the amount of Ag and N elements on the surface of BC5 increases with the increase of the AgNO_3_ precursor concentration during the dipping process, indicating that more precursor is adsorbed on the sample for higher precursor concentrations, but not all of it is reduced to form NPs.

It is worth noting that the different concentrations of the Ag precursor do not necessarily lead to the formation of more AgNPs in the BC5. Indeed, as shown in Figures 2a and S10b of the Supporting Information, for all treated samples, the overall weight loss is 5–7% lower compared to the uncoated samples. Therefore, we may conclude that the AgNP’s maximum amount in the composite structure should range between 5 and 7 wt %. Based also on the fact that the NPs formed have similar structural characteristics, as previously discussed, we can conclude that the different concentrations used in this study do not affect significantly the properties of the formed NPs. Therefore, from now on, we will focus only on one of the AgNPs’ coated samples (i.e., BC5AgNPs_0.1M_).

To investigate the functional groups and any possible chemical interactions between the polymer matrix and the fillers, the ATR-FTIR analysis of the uncoated and AgNPs coated samples is performed, as shown in Figures 2c and S12. The spectrum of the BC0 presents all the characteristic bands of the PVA/PVP composite, such as the broad peak at 3283 cm^–1^, which is assigned to the PVA’s O―H stretching vibration and the adsorbed humidity. The bands at 2939, 2918, and 1657 cm^–1^ are attributed to the asymmetric and symmetric stretching of C―H and the carbonyl group (C=O) of PVP,^56^ respectively. The peaks at 1424 and 1090 cm^–1^ correspond to the C―H bending and C―O stretching vibration, while the band at 1289 cm^–1^ can be related to the C―N stretching vibration of PVP.^55^ When the CB filler is introduced (sample BC5), no significant modifications of the spectrum are observed, apart from the appearance of the peak at 1576 cm^–1^ characteristic of the C=O stretching vibration of the CB filler (Figure S12 and Table S4) and the broadening of the O―H peak, indicating that there are no specific interactions between the two components. For the AgNP-coated BC5, the spectrum is similar to that of the BC5; nonetheless, a shift of the O―H peak toward lower wavenumbers is observed (from 3283 to 3265 cm^–1^), indicating possible hydrogen bonds with the Ag component.^57^

**Figure 3:**
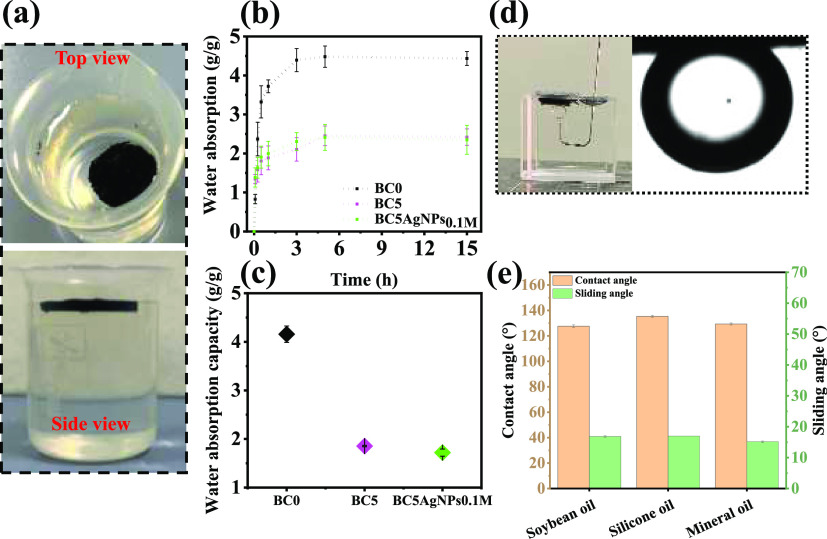
(a) Photographs of top and side views of BC5 floating on MilliQ water for 5 months, proving the self-floating ability and stability. (b) The water absorption capacity of the samples in different time intervals until 15 h. (c) Water absorption capacity at equilibrium. (d) Photograph of the BC5 demonstrating underwater oleophobicity. (e) Underwater oil contact angle and sliding angle using different types of oils on the BC5.

Normally, the solar evaporation process is linked with the transportation of water from the bulk water to the top surface of the evaporator. As shown in Figure S13 of the Supporting Information, immediately after the deposition of a droplet on the BC0 surface, the water contact angle is ca. 57° indicating a hydrophilic surface. However, the water droplets deposited on the BC5 and BC5AgNPs samples are immediately absorbed, indicating that the different pore structures and surface roughness of these samples significantly enhance their hydrophilicity. Exceptionally, the wet BC5 and BC5AgNPs samples have a self-floating ability on water, which is preserved even after leaving the samples for ca. 5 months in contact with water at room temperature (Figure 3a). The dynamic water uptake of the composite cryogels was also explored, and it was found that the highest rate of water uptake occurs in the first 30 min, while the equilibrium is reached within 5 h (Figure 3b). On the other hand, BC0 takes up water faster, with the maximum value reached (4.16 ± 0.17 g g^–1^) to be higher than those of the BC5 (1.85 ± 0.01 g g^–1^) and BC5AgNPs_0.1M_ (1.72 ± 0.07 g g^–1^) (Figure 3c) indicating that the addition of the CB filler into the polymer matrix affects the polymer chains mobility and also reduces the overall porosity of the BC0 as previously discussed, both resulting in the reduction of its ability to absorb water.

BC5 is also underwater oleophobic (Figure 3d), as the water saturated-sample repels the oil droplet from its surface. For a more detailed analysis, the underwater oil contact angles of BC5 for different oils (i.e., soybean, silicone, and mineral oil) are presented in Figure 3e. For all oils tested, the underwater oil contact angle is ca. 134° with a sliding angle below 20°. It should be noted that the volume of oil drops remained the same in the experiment’s time frame during contact with the sample, and no residue of oil remained on the surface of the sample after the sliding angle tests. Consequently, when the BC5 is saturated with water, it creates a strong interfacial layer of oil/water, which hinders oil trapping, making the material oleophobic.^58^ Such an oil antifouling property guarantees the efficient interaction of water with the material, even in the presence of oil, which is beneficial for applications dealing with photothermal desalination or purification processes.

**Figure 4:**
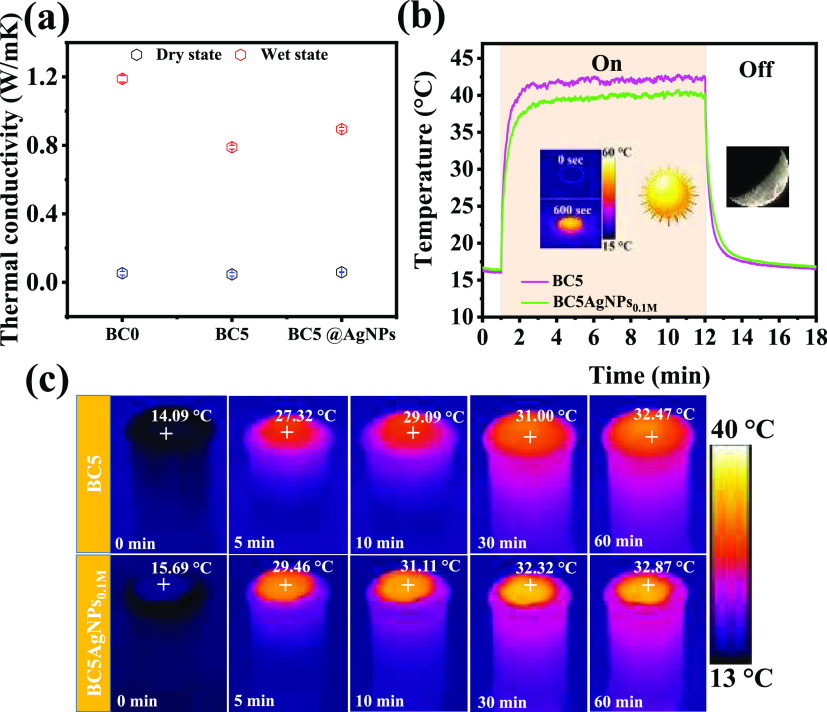
(a) Thermal conductivity of the cryogels. (b) Temperature profiles of BCs in dry conditions under 1 Sun irradiation (on) and after switching of the lamp (off). Inset: IR images of a representative sample. (c) IR images of the BC samples when they were floating on the bulk water under 1 Sun irradiation for 1 h.

The basic requirements for efficient solar steam generation through a porous material are the high absorption of the solar light in the whole spectrum and low thermal conductivity. As shown in Figure S14, BC5 exhibits on average more than 85% of absorbance in the NIR and almost 100% in the UV–vis range. This is attributed to the presence of the CB, which, apart from its intrinsic optical properties, also induces enhanced surface roughness compared to the smoother surface of BC0, which helps in the increase of optical absorbance and a decrease in reflectance.^59,60^ BC5 also exhibits lower thermal conductivity in the dry state (ca. 0.0595 W m^–1^ K^–1^) compared to BC0, while the presence of AgNPs does not significantly affect this value (Figure 4a). The lower thermal conductivity can be attributed to the overall higher pore size,^61^ responsible for a higher amount of air traps and scattering of phonons.^62^ As expected, in the wet state, the thermal conductivity of the samples increases with BC0 reaching 1.188 W m^–1^ K^–1^, whereas BC5 and BC5AgNPs_0.1M_ reach values of 0.789 and 0.894 W m^–1^ K^–1^, respectively. This can be explained by the fact that the BC0 absorbs a higher amount of water; as previously proved, contributing to the higher thermal conductivity in the wet state compared to the BC5 samples.

The photothermal properties, such as surface temperature and heat localization within the samples under solar irradiation, were monitored by an IR camera. The surface temperature of the BC5 and BC5AgNPs_0.1M_ in dry conditions rises sharply within the first 2 min of irradiation and then reaches a steady state temperature with a maximum of 42.54 and 40.25 °C, respectively (Figure 4b). Also, in this case, it can be confirmed that the presence of the AgNPs does not influence the performance.

When samples float on water, the IR thermal imaging under solar irradiation (1 Sun) reveals that both BC5 and BC5AgNPs_0.1M_ display a sharp increase of the surface temperature in the first 5 min, (Figure 4c) which is c.a. 13–14 °C higher than that of the sample’s initial temperature, while after 1 h of irradiation, the temperature stabilizes at higher values, with a final increase of 18.38 and 17.18 °C for the BC5 and BC5AgNPs_0.1M_, respectively. On the other hand, the bulk water is not significantly affected, indicating that most of the heat is localized in the samples rather than in the surrounding environment. This, combined also with the other properties of the BCs, such as their hydrophilic network, porous structure, broad optical absorbance, and low thermal conductivity, recommend that such materials have favorable features for biocomposite solar steam generators.

**Figure 5:**
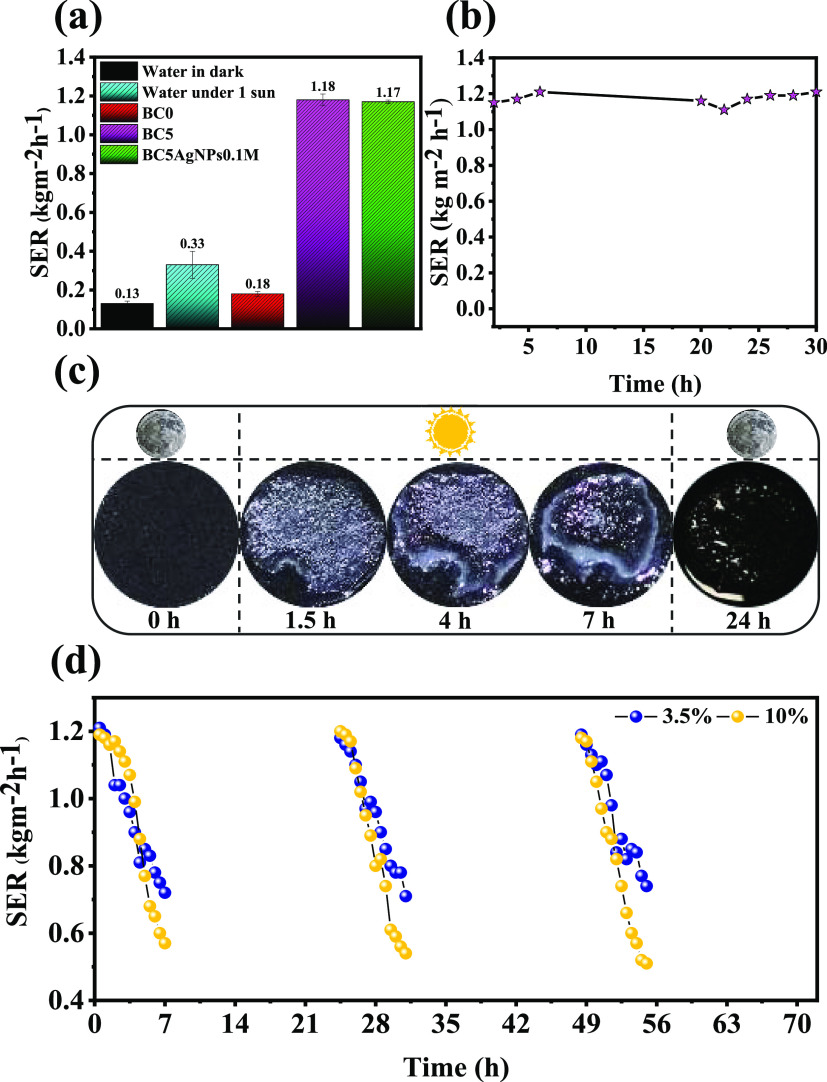
(a) SER in the dark and under 1 Sun irradiation of the different cryogels and of the plane water. (b) SER of BC5 monitored while floating on water for 30 h of continuous solar irradiation (1 Sun). (c) Salt antifouling performance of the BC5 floating on artificial seawater (3.5 wt %) under solar irradiation (1 Sun for 7 h) and in the dark (17 h). The photographs show the evolution of the salt accumulation on the surface of the sample. (d) Long-run SER of BC5 floating on artificial seawater (3.5 and 10 wt %) for 3 cycles of 1 Sun irradiation for 7 and 17 h in the dark.

To determine the efficiency of the photothermal materials, the SER was measured under 1 Sun irradiation. To do so, BCs were allowed to float on water, and the SER was measured by monitoring the cumulative mass loss of water due to evaporation. Supporting Information, Figure S15 displays the net water mass change driven by evaporation, which in the presence of the samples occurs much faster than that of water in the absence of the samples. In particular, the SER of only water under 1 Sun irradiation is 0.33 kg m^–2^ h^–1^, while the SER of BC5 (1.18 kg m^–2^ h^–1^) and BC5AgNPs_0.1M_ (1.17 kg m^–2^ h^–1^) is 3.57 and 3.54 times higher (Figure 5a). At the same time, the solar-to-vapor conversion efficiency was calculated to be 81.97 and 81.11% for BC5 and BC5AgNPs_0.1M_, respectively, and such performance is in the same range as the other PVA-based photothermal materials (Table S5). Exploring the prolonged photothermal capability, with 30 h of solar irradiation (Figure 5b), it can be concluded that the SER of the cryogel is not altered significantly, confirming the stability of the performance of the developed material, and indicating the possibility to be used as a valuable component in continuous irradiation environments.

To investigate the ability of the developed materials for solar desalination applications, the BC5 was placed on two different artificial seawater solutions with a salt concentration of 3.5 and 10 wt %. The surface of BC5 monitored at different time intervals under 1 Sun irradiation is presented in Figure 5c. Due to the diffusion of the salty water into the sample, salt crystallization is observed on the surface of the sample, with crystals appearing after 1.5 h of interaction (Figure S16). After 7 h of 1 Sun irradiation, the sample is highly loaded with salt crystals, which start to disappear during the dark period, and after 17 h, no dreg is left on the surface of BC5. This is attributed to the enhanced wettability of the cryogel, which leads to the salt sediments’ redissolution into the bulk water when evaporation is halted. Such a self-cleaning ability of the sample, i.e., the dissolution of the accumulated salt on the surface of BC5 during the night-time, confirms the salt transport from the top surface of BC5 to the bottom toward the bulk brine.^63^

The evaporation rate for prolonged utilization of the photothermal sample was monitored for 3 days of sequential solar irradiation and dark for the water of both salinities. As shown in Figure 5d, during the 7 h of solar irradiation, the evaporation rate reduced from ∼1.19 to 0.76 kg m^–2^ h^–1^ and from ∼1.22 to 0.53 kg m^–2^ h^–1^ in the case of 3.5 and 10 wt % salt water, respectively, because of the salt deposition on the cryogel’s surface which disturbs the light absorption and enhances its reflection.^64^ The SER declination was more evident when the sample floated on the brine with 10 wt % salinity due to a greater salt concentration, which resulted in more accumulated salt deposition on the sample’s surface. Nonetheless, in both cases, under darkness, most of the salt residues were dissolved, resulting in the regeneration of the solar evaporator. In the second and third cycles of solar irradiation, the performance is similar to that in the first cycle, ensuring (1) the recovery of the evaporation rate in continuous conditions, (2) the high stability for solar desalination performance, promising for realistic applications, and (3) the necessary antifouling properties.^64^

**Figure 6:**
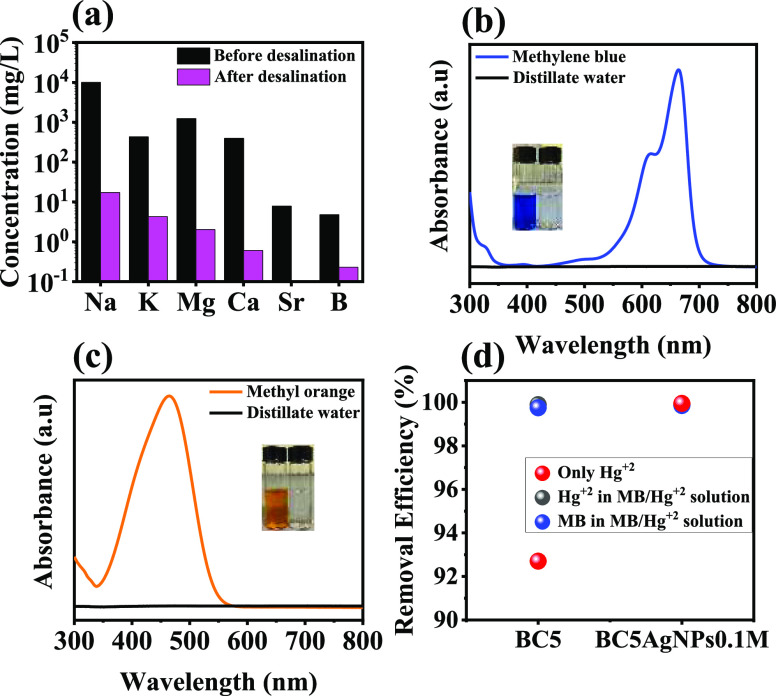
(a) Concentration of the main elements in synthetic seawater (3.5 wt %) before and after desalination, determined by ICP-OES. (b) MB and (c) MO dye solutions before and after solar purification. (d) Removal efficiency of BC5 or BC5AgNPs_0.1M_ floating on wastewater solution under 1 Sun for 7 h for Hg^+2^ loaded wastewater (10 mg L^–1^, red cycles) and simulated wastewater (i.e., 10 mg L^–1^ Hg^+2^ and 30 mg L^–1^ MB, gray and blue cycles, respectively), measured by ICP-OES and UV–vis spectrophotometer.

Analyzing the vapor collected after the solar evaporation in the presence of synthetic seawater (3.5 wt %) (Figure S17), it was found that the concentration of the elements in the collected distillate was substantially decreased, reaching an overall desalination efficiency of 98.92% (Figure 6a) and ion concentration far below the limit for drinking water set by the World Health Organization (WHO).^65^

Therefore, the developed material presents a high ability for desalination and enhanced stability over salt fouling. However, seawater does not only contain salt elements but also various pollutants depending on the geographical area where the desalination occurs. Therefore, for an efficient photothermal desalinator, also the ability to collect pure water from polluted areas should be explored. Although the developed cryogels can effectively interact and remove organic and inorganic pollutants in static conditions, as described in the Supporting Information, Section S1, the biocomposite cryogel’s ability to collect pure water from water with both inorganic and organic pollutants through the photothermal action was further explored.

In particular, the distillate collected through the cryogel after solar evaporation from a source solution of simulated wastewater containing organic dyes such as MB (30 mg L^–1^) and MO (30 mg L^–1^) was analyzed, and as shown in Figures 6b,c, no dyes are present in the distillate, while in the water source, the absorption spectra indicate the presence of both types of dyes (Figure S18). Also, the performance of the cryogel after solar evaporation of an aqueous solution of Hg^+2^ ions was investigated, with the distillate collected upon the solar evaporation process to confirm that the cryogels can effectively exclude the presence of the Hg^+2^ ions with an efficiency of ∼99.0% (Figure 6d). To further evaluate the cryogel’s performance in multicomponent wastewater, the analysis of the distillate after the solar evaporation starting from an aqueous solution containing both Hg^+2^ and MB was performed. As shown in Figures 6d and S19, the removal efficiency of both Hg^+2^ and MB was higher than 98%, proving the effectiveness of the developed materials for photothermal purification applications.

**Figure 7:**
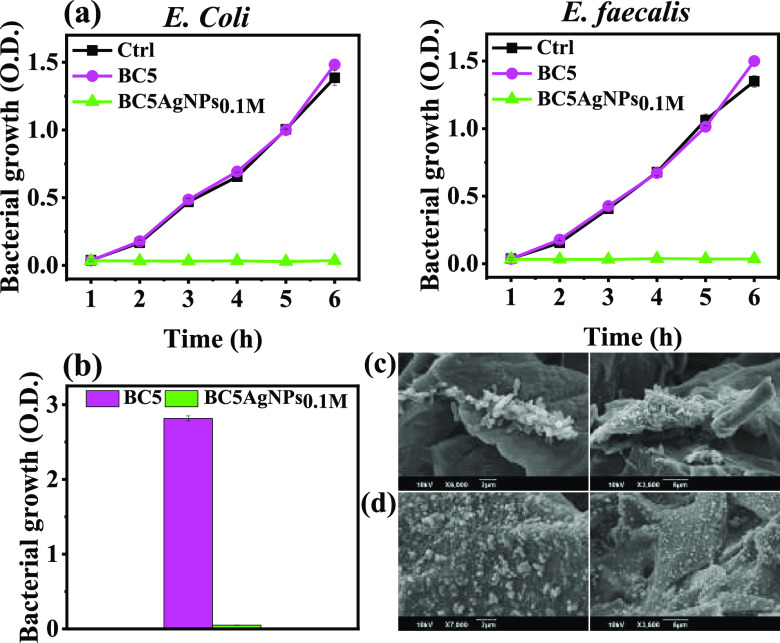
(a) OD_600_ measurements of *E. coli* and *E. faecalis* growth in the presence of BC5 and BC5AgNPs_0.1M_. Ctrl represents bacterial suspension without samples. (b) OD_600_ measurements of *E. coli* growth on BC5 and BC5AgNPs_0.1M_ after 1.5 h of solar irradiation. SEM analysis of (c) BC5 and (d) BC5AgNPs_0.1M_ after their interaction with bacteria-loaded media.

However, real wastewater not only contains organic and inorganic pollutants together with the salt ions but also various microorganisms such as bacteria, which eventually may attach and grow to the surface of the solar evaporators affecting their performance.^66^ To explore the interaction of the developed cryogels with bacteria, their antibacterial performance against representative Gram-negative and Gram-positive bacterial species was evaluated by using *E. coli* and *E. faecalis*, respectively. As shown in Figure 7a, the BC5 does not exhibit any antibacterial effect, while the AgNP-coated sample demonstrates an efficient inhibition of bacterial growth even after 5 h of incubation, both on *E. coli* and *E. faecalis* species, demonstrating a wide-range effect on different kinds of bacteria. To further verify the antibacterial activity of the BC5AgNP_0.1M_, overnight bacterial growth in solid culture plates was performed, using as starting solution the bacterial liquid culture resulting from the previous experiment (Figure S20). No bacteria were detected in the samples, demonstrating the complete antibacterial effect even after the long incubation period.

To explore the anti-biofouling properties of the developed system and to further investigate the validity of water vaporization, the SER was monitored while BC5 and BC5AgNPs_0.1M_ were floating separately on a bacterial suspension (*E. coli*) under 1 Sun irradiation. The calculated SER values reveal that the performance is not affected by such biological conditions (SER: 1.19 and 1.21 kg m^–2^ h^–1^ for BC5 and BC5AgNPs_0.1M_, respectively) (Figure S21). After exposure, the samples were incubated overnight in LB medium, and the bacterial growth was then monitored with OD_600_ measurements (Figure 7b). Interestingly, the BC5 sample showed considerable bacterial growth, while, conversely, the BC5AgNPs_0.1M_ demonstrated excellent antibacterial capability, indicating that the sample after the photothermal performance does not contain any bacterial population on its surface. This is also confirmed by the SEM images (Figure 7c), where the presence of a high amount of intact bacteria grown on the BC5 contrary to the BC5AgNPs_0.1M_ is shown (Figure 7d), demonstrating the bacterial antifouling properties driven by the presence of the AgNPs (Figure S22).

## Conclusions

In summary, a silver-coated PVA-based vegetable waste biocomposite cryogel with high stability, stable performance, and multifunctional properties was developed, able to purify water through a photothermal action, with salt and bacteria antifouling properties. As proved, the biocomposite cryogel presents a highly porous structure, sunlight absorption throughout the spectrum, appropriate wetting properties, self-floating ability, low thermal conductivity, and prolonged stability in water resulting in a solar-to-vapor conversion efficiency of 81.11% under 1 Sun irradiation. Under the photothermal action, the biocomposite cryogel can purify water from dyes and mercury ions with an efficiency of >99%. In addition, the anti-salt-fouling and the anti-biofouling properties guarantee that the developed biowaste-based cryogel can open new perspectives for wastewater treatment.

## Supporting Information Available

Image and particle size distribution of carbonized beetroot powder; photographs of the cryogel before and after freeze-drying and the fabrication procedure scheme; top surface morphology of BCs; mechanical analyses of BC5; comparison of the compressive stress with other materials; MIP of BCs; Raman spectra of BCs; morphological analyses and EDX of AgNP-coated samples; XRD and TGA analyses of BCs; XPS and FTIR spectra of BCs; photographs of the water contact angle on the BCs; optical absorption of BC5; mass change due to water evaporation of BCs; comparison of cryogel performance with previous studies; optical microscopy images of salt accumulation on the BC surface; UV–vis absorption spectra of dyes; antibacterial tests and SER; and MB dye and Hg removal analysis in static conditions (PDF)
