# Supplementary material for: Biocomposite Cryogels for Photothermal Decontamination of Water
Source: Langmuir. 2023 May 26;39(22):7793–803. doi: 10.1021/acs.langmuir.3c00623 (PMC10249410; doi:10.1021/acs.langmuir.3c00623)
Supplement: Supplementary file 1 — la3c00623_si_001.pdf [file la3c00623_si_001.pdf]

Biocomposite Cryogels for Photothermal Decontamination of Water

Muhammad S. Zafar,[†,‡] *Francesca Gatto,*[#] *Giorgio Mancini,*[†] *Simone Lauciello,*[§] *Pier P. Pompa,*[#] *Athanassia Athanassiou,*[†] *and Despina Fragouli,*[†,*]

[†]Smart Materials, Istituto Italiano di Tecnologia, via Morego 30, 16163 Genova, Italy

[‡]Dipartimento di Informatica, Bioingegneria, Robotica e Ingegneria dei Sistemi (DIBRIS), Università degli Studi di Genova, Via Opera Pia 13, 16145 Genova, Italy

[#]Nanobiointeractions & Nanodiagnostics, Istituto Italiano di Tecnologia, Via Morego 30, 16163 Genova, Italy

[§]Electron Microscopy Facility, Istituto Italiano di Tecnologia, via Morego 30, 16163 Genova, Italy

Corresponding author: despina.fragouli@iit.it

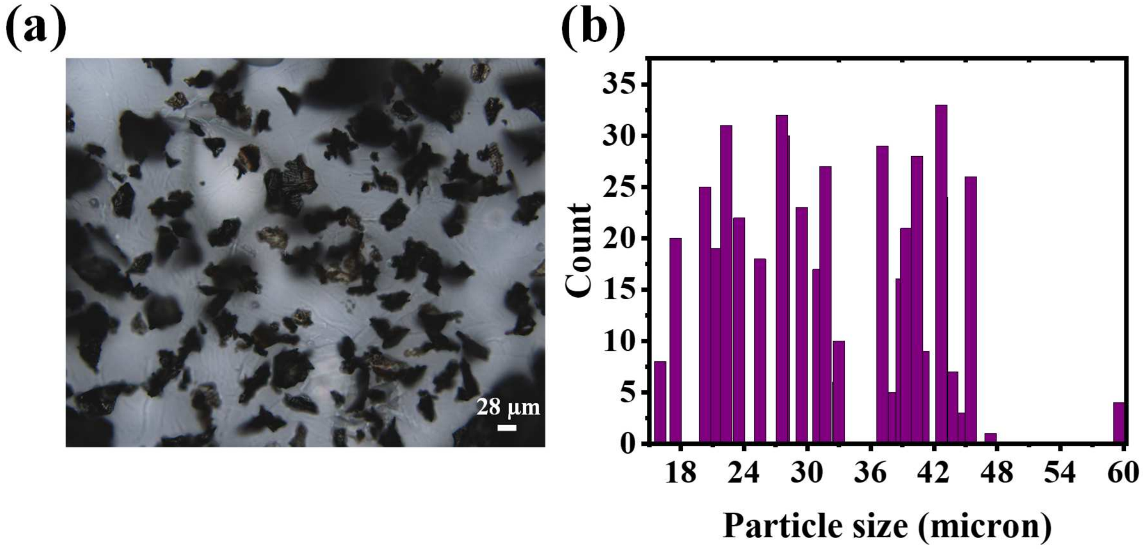

Figure S1. (a) Image of CB powder. (b) Particle size distribution of CB powder. Images were captured using a profilometer and 33 different particles were considered for the size distribution analyzed by the ImageJ software.

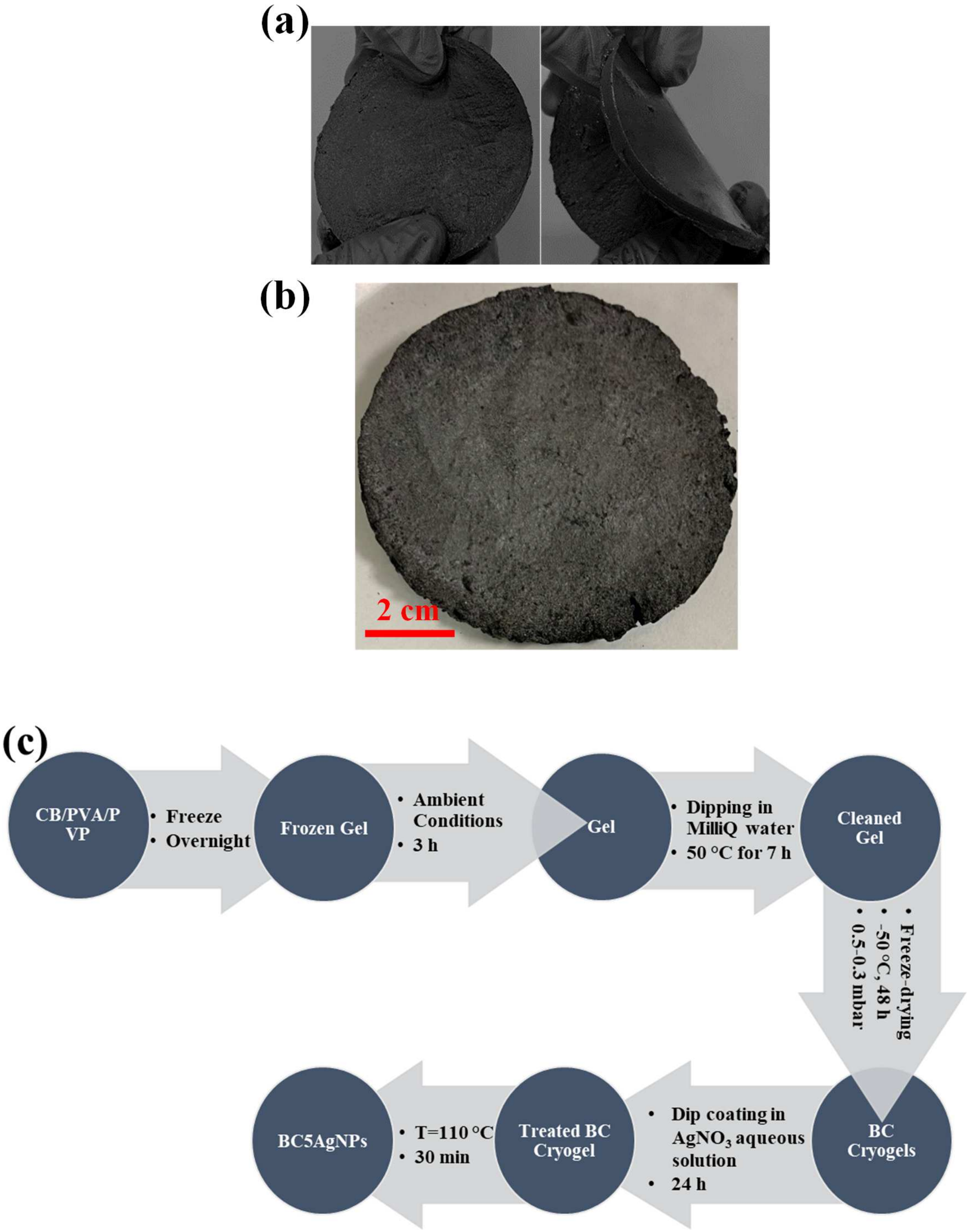

Figure S2. Photographs of the BC5 cryogel (a) before and (b) after freeze-drying. (c) Flow chart of the experimental process for the preparation of the AgNPs coated BC cryogels.

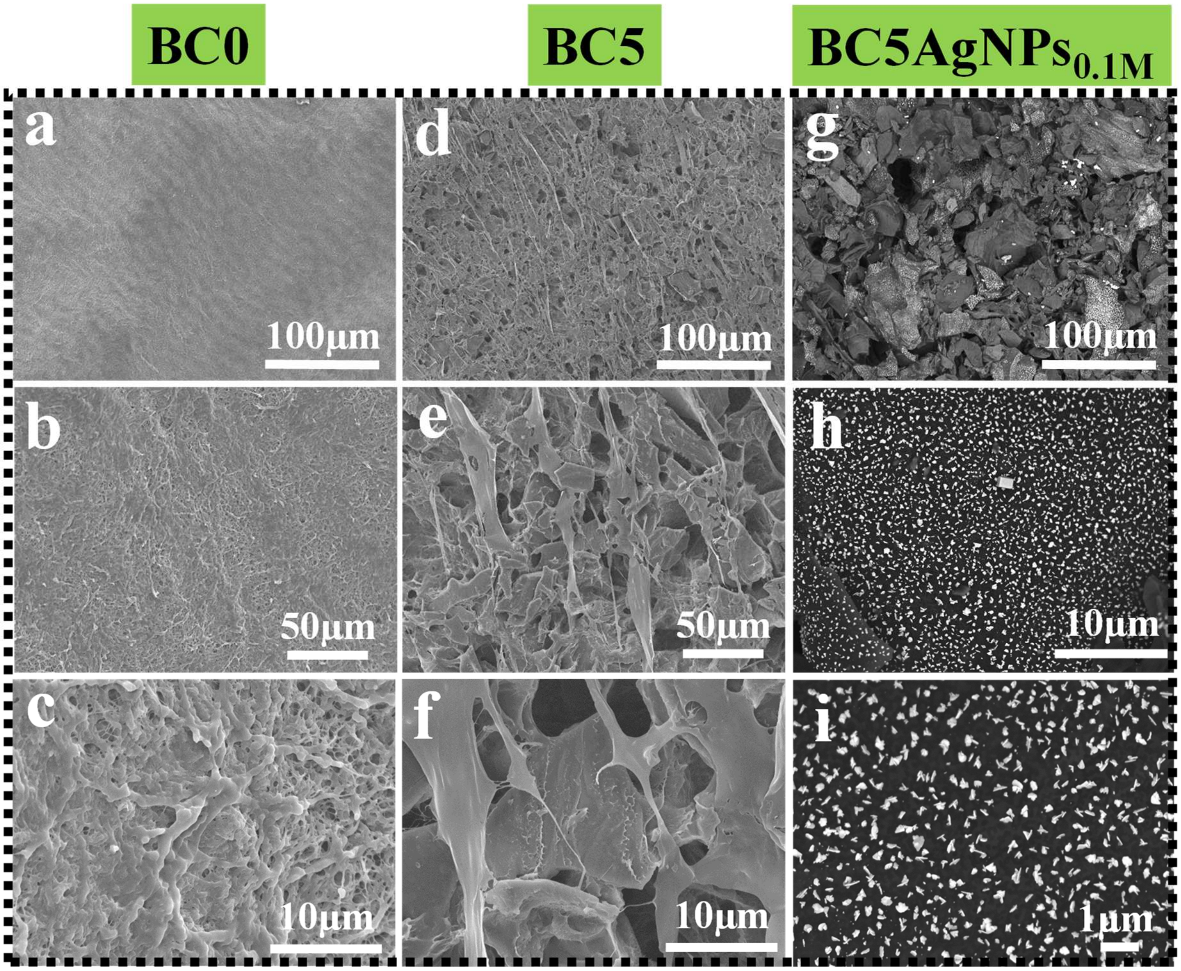

Figure S3. Top surface SEM images of the BC0 (a-c) (d-f) BC5 and (g-i) BC5AgNPs$_{0.1M}$ in different magnifications.

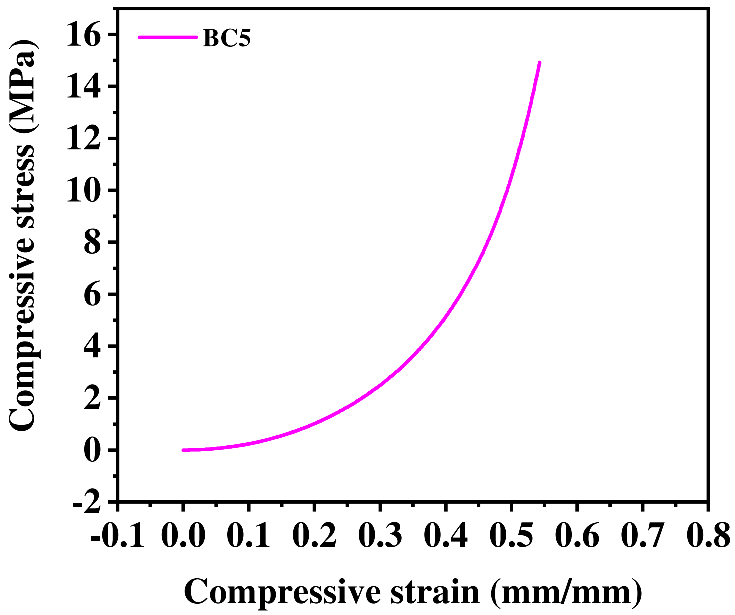

Figure S4. Compressive strength of BC5. For the measurement, a round shape sample with thickness of 2.5 mm and 8 mm of diameter was used. The compressive test was performed using an INSTRON 3365 (USA) instrument with dual column table top universal testing system. The rate of moving plunger was 1 mm/min. The compressive modulus was determined to be c.a. 6.9 MPa.

Table S1. Comparison of representative stress values of solar evaporators

**Sample**	**Compressive Stress (MPa)**	**Reference**
conjugated microporous polymer (CMP) aerogel	0.54	[1]
ATP (Attapulgite) based PArG aerogel	0.006	[2]
Kapok fiber-Polypyrrole aerogel	0.12	[3]
Attapulgite- polyvinyl alcohol-nanofibrillated cellulose (APN) aerogel	0.28	[4]
Blank hollow spacer fabric (BHSF)	0.75	[5]
Polypyrrole-hollow glass microspheres-melamine sponge (PPy-HGMAM)	0.95	[6]
Lignocellulose-based double-layered hydrogel (LC@LCG)	0.04	[7]
Polyvinyl alcohol-agarose-Carbon nanotube based aerogel	10	[8]
Semiconductive in situ-polymerized MnO_2 nanowires/chitosan (SPM-CH) hydrogel	0.42	[9]
Phenolic aldehyde foams (PAFs)	0.07	[10]
Polydimethylsiloxane/carbon nanotubes (PDMS/CNTs) modified melamine	0.04	[11]
Polypyrrole modified sugarcane (PPy-SC) based evaporator	0.17	[12]
Hierarchically nanostructured gel (HNG)	0.35	[13]
Poly (2-acrylamido-2-methylpropanesulfonic acid - polyacrylamide (PAMPS–PAAm) gel	17.2	[14]
Pine wood	26.1	[15]
Polyvinyl alcohol/Starch/iodine hybrid (PSIG) hydrogel	1	[16]
Geopolymer–biomass mesoporous carbon composite (GBMCC)	35	[17]
Cellulose hydrogel	14.5	[18]
Fungal hypha activated carbon pad (FhACPad)	2.25	[19]
Cotton fiber based polyurethane Foam	0.87	[20]
Graphene oxide/Chitosan (GO/CH) aerogel	16.98	[21]
**Biocomposite cryogel**	**14.92**	**This work**

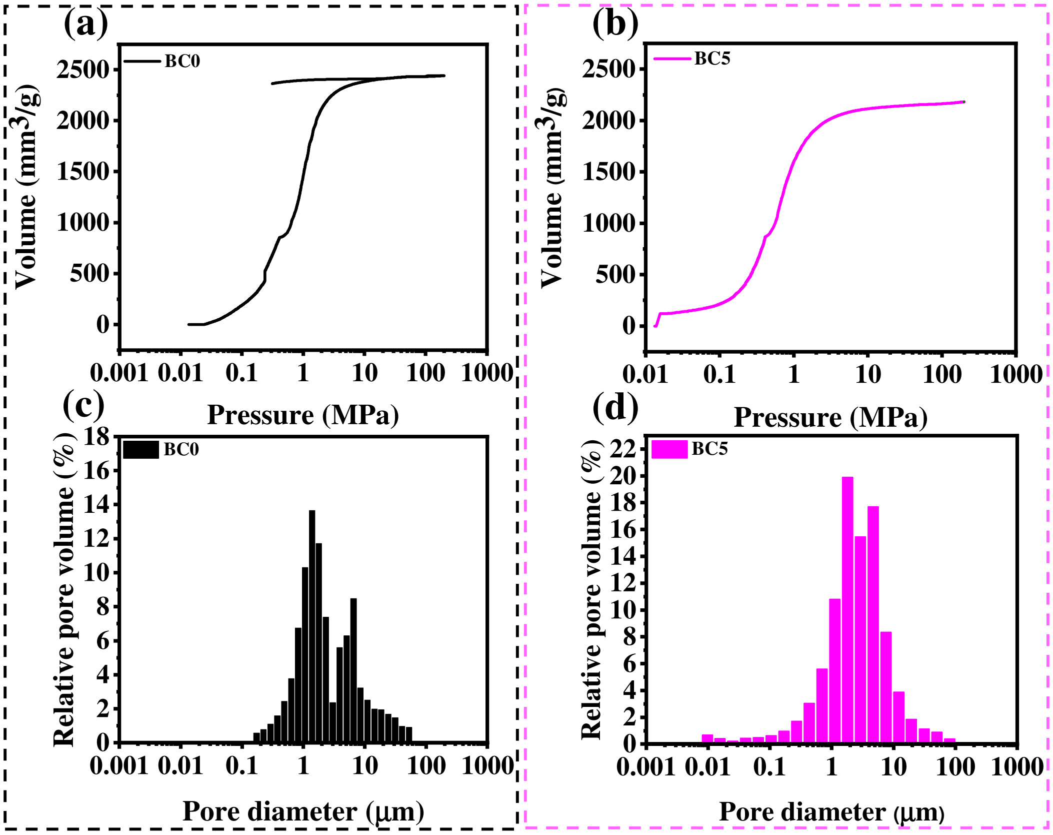

Figure S5. Mercury intrusion-extrusion and pore size distribution profile of (a,b) BC0 and (c,d) BC5.

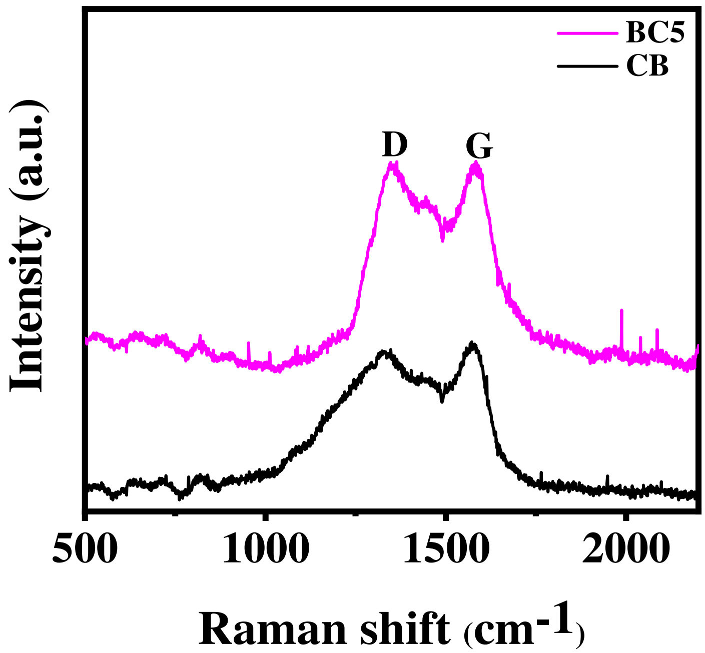

Figure S6. Raman spectra of CB and BC5.

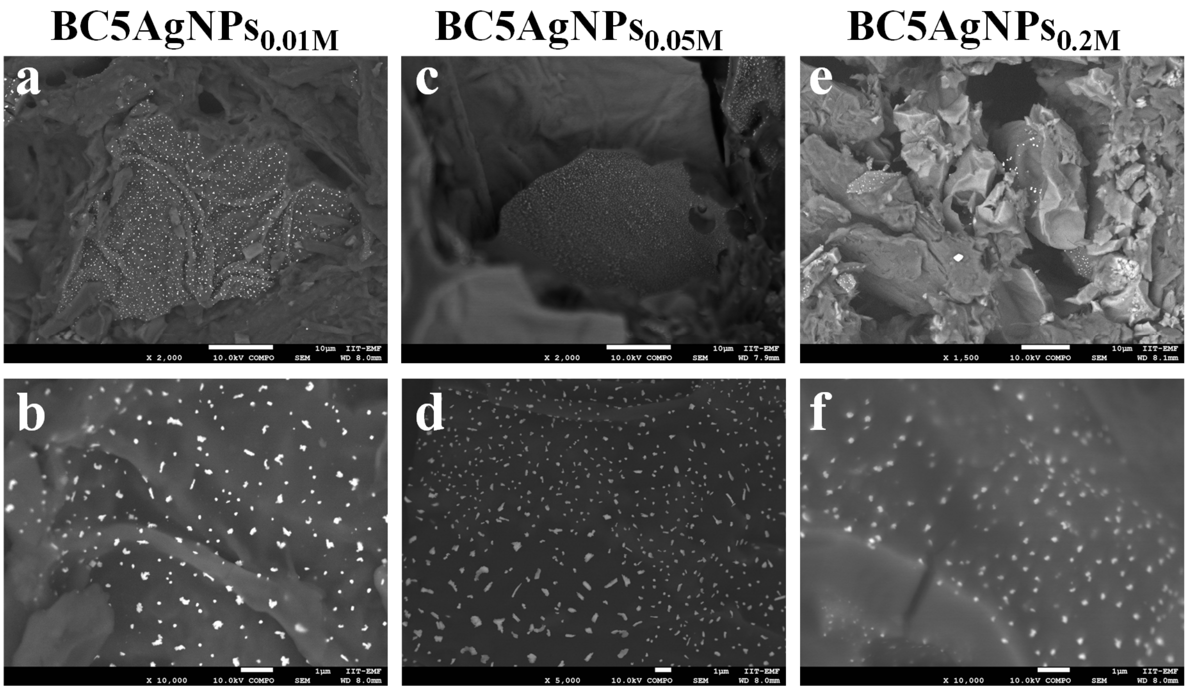

Figure S7. SEM images of BCs decorated with AgNPs (a-b) $BC5AgNPs_{0.01M}$ (c-d) $BC5AgNPs_{0.05M}$ (e-f) $BC5AgNPs_{0.2M}$.

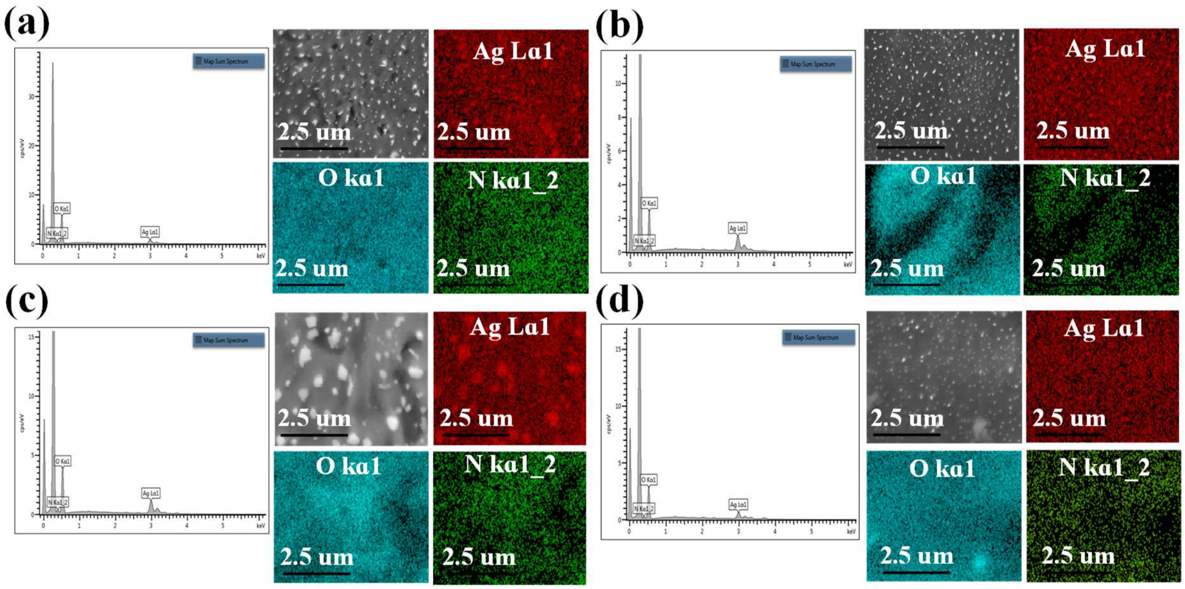

Figure S8. EDX-SEM analyses of BC5 coated by $AgNO_3$ at different concentrations (a) 0.01M (b) 0.05M (c) 0.1M (d) 0.2M.

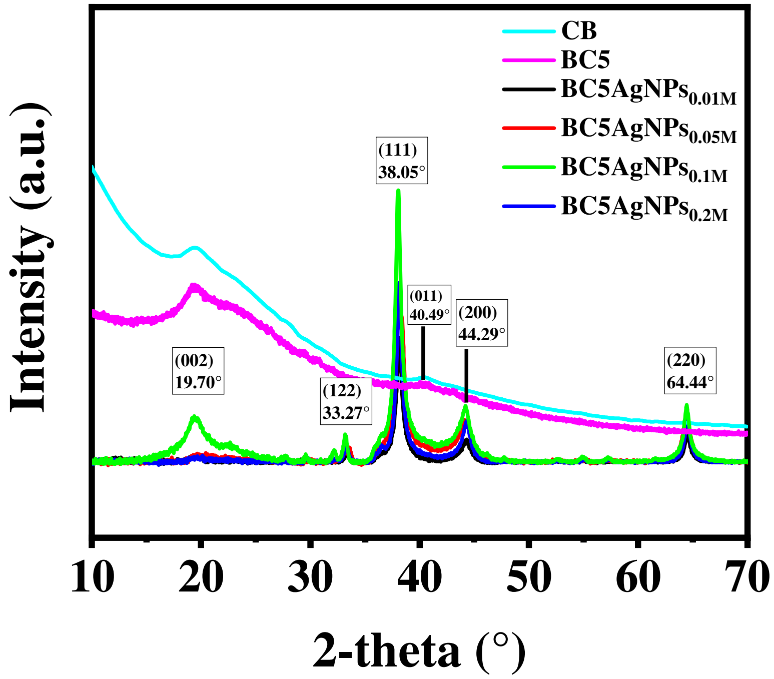

Figure S9. (a) XRD analyses of the cryogels. The crystalline size of AgNPs was calculated using the Debye-Scherrer equation (4). The peak at 38.03° of the Ag loaded samples was used to analyze the particle crystalline size.

$$n = \frac{K\lambda}{\beta cos\theta}$$

Here, value of K (shape factor) is 0.9 to 1. λ is wavelength of x-ray with the value of 1.54 Å. β is full width at half height and θ (19°) is the Bragg angle. The average crystallite size of 10.48 nm, 11.75 nm, 8.85 nm and 10.65 nm for $BC5AgNPs_{0.01M}$, $BC5AgNPs_{0.05M}$, $BC5AgNPs_{0.1M}$, $BC5AgNPs_{0.2M}$ respectively.

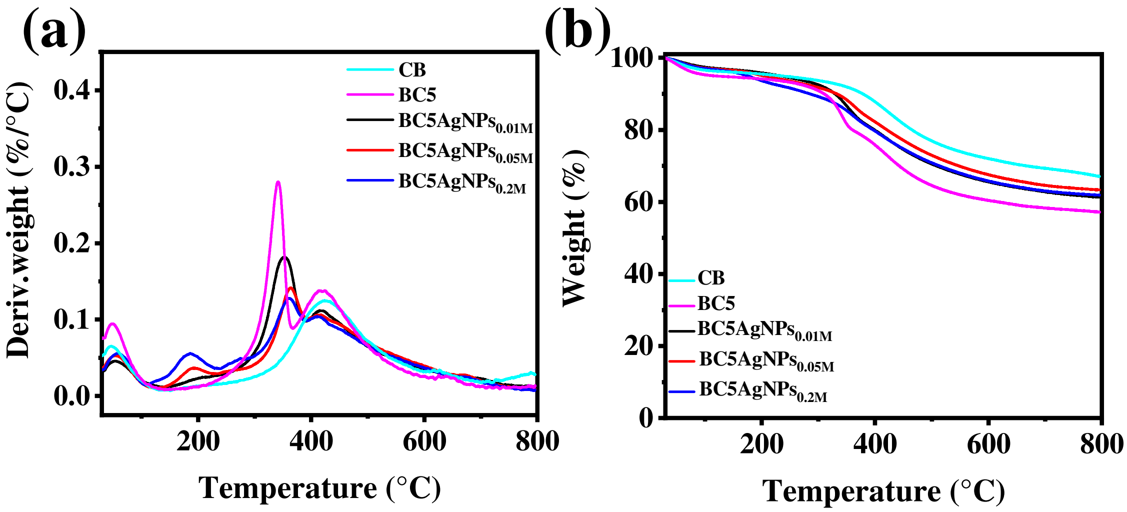

Figure S10. (a) DTGA and (b) TGA of CB filler and of the different cryogels.

Table S2. T_{max} at each point for the BCs

Samples	T_{max} (°C)	T_{max} (°C)	T_{max} (°C)	T_{max} (°C)
BC0	**80**		**260**	**415**
BC5	**50**		**341**	**420**
BC5AgNPs$_{0.01M}$	**55**		**350**	**420**
BC5AgNPs$_{0.1M}$	**58**	**190**	**365**	**415**
BC5AgNPs$_{0.2M}$	**60**	**185**	**360**	**410**
CB	**50**			**424**

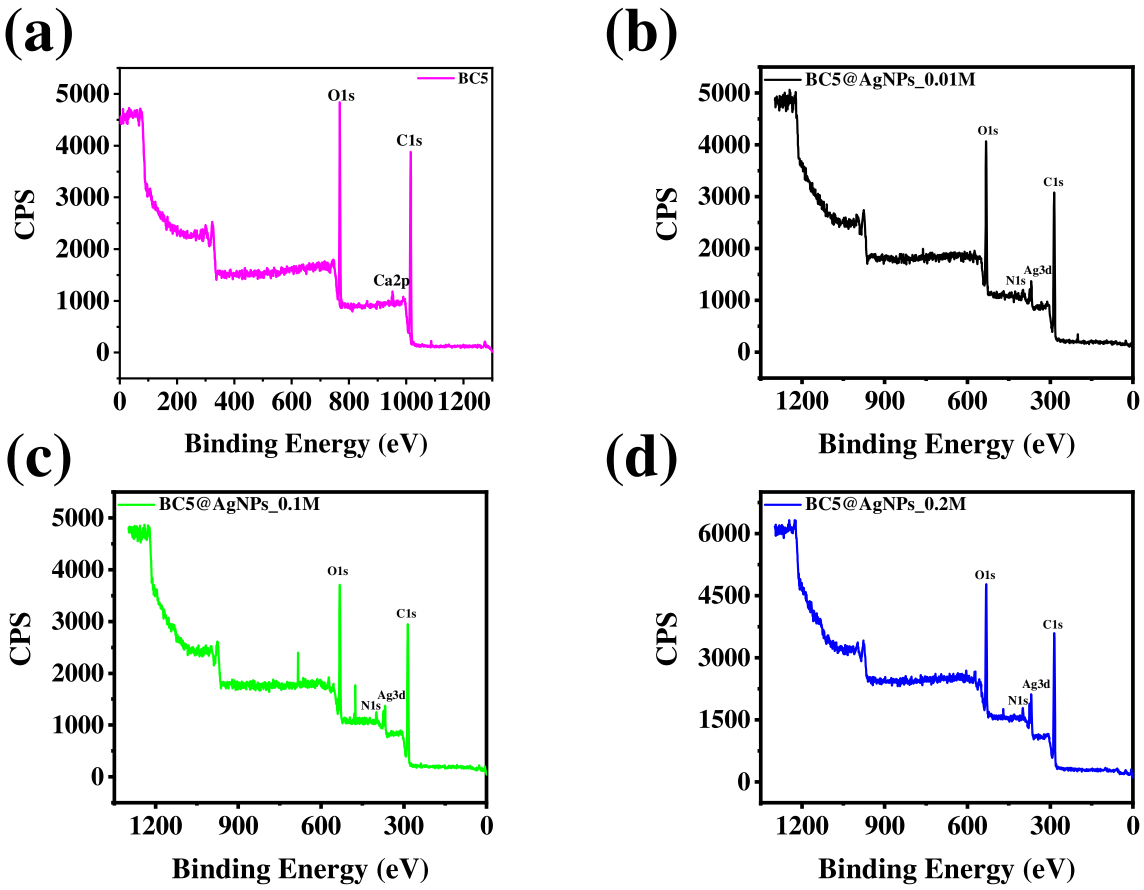

Figure S11. XPS spectra of (a) BC5 (b) BC5AgNPs$_{0.01M}$ (c) BC5AgNPs$_{0.1M}$ (d) BC5AgNPs$_{0.02M}$.

Table S3. Atomic percentage of BCs

	C1s	*O1s*	*N1s*	*Ag3d*	*Ca2p*
BC5	72.80	26.14			1.04
BC5AgNPs$_{0.01M}$	71.86	26.00	1.08	1.05	
BC5AgNPs$_{0.1M}$	72.55	24.16	1.84	1.43	
BC5AgNPs$_{0.2M}$	72.18	23.63	2.02	2.14	

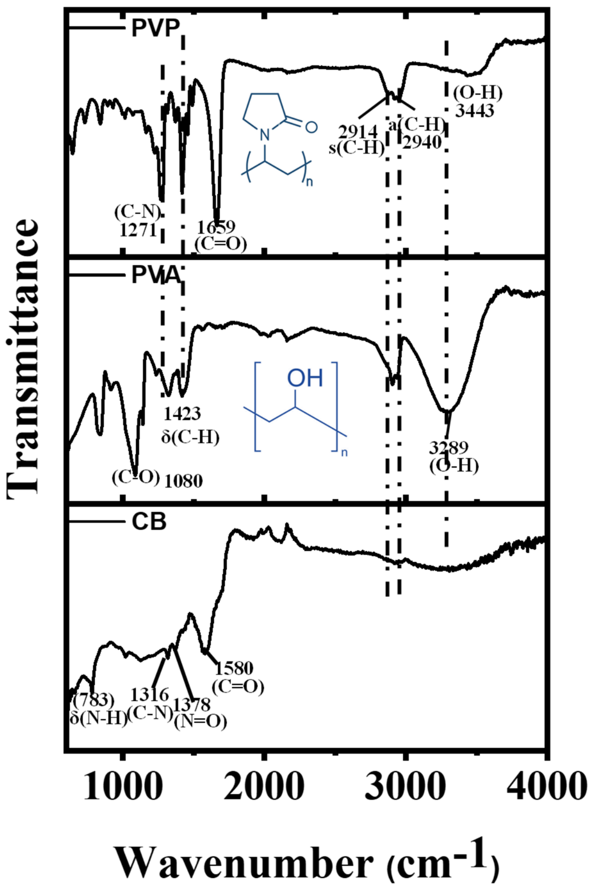

Figure S12. FTIR spectra of pristine PVP, PVA powder and CB filler.

Table S4. Wavenumbers and functional groups associated to pure PVA, PVP and CB filler

Sample type	Bands (cm^{-1})	Chemical Associated groups
**PVA**	3272	O-H Stretching
	2940	C-H Asymmetric Stretching
	2907	C-H Symmetric Stretching
	1418	C-H Bending
	1088	C-O Stretching
**PVP**	3393	O-H Stretching
	2955	C-H Asymmetric Stretching
	2892	C-H Symmetric Stretching
	1424	C-H Bending
	1289	C-N Stretching
**CB**	3346	O-H Stretching
	2938	C-H Asymmetric Stretching
	2916	C-H Symmetric Stretching
	1580	C=O
	1378	N=O Stretching
	1316	C-N Stretching
	783	N=H bending

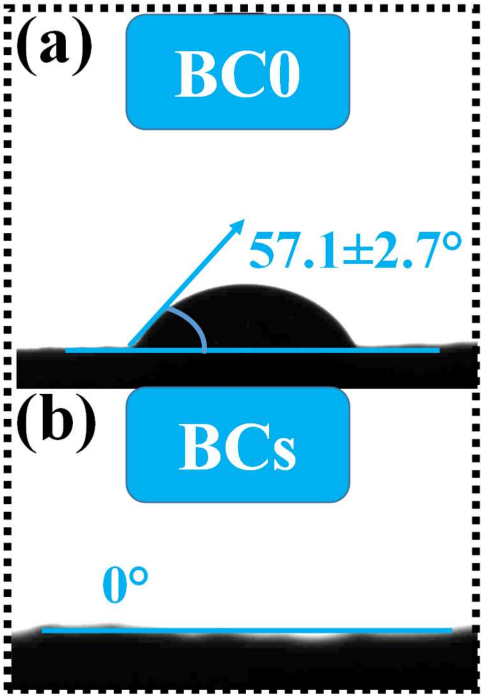

Figure S13. Water contact angle of (a) BC0 and (b) BCs.

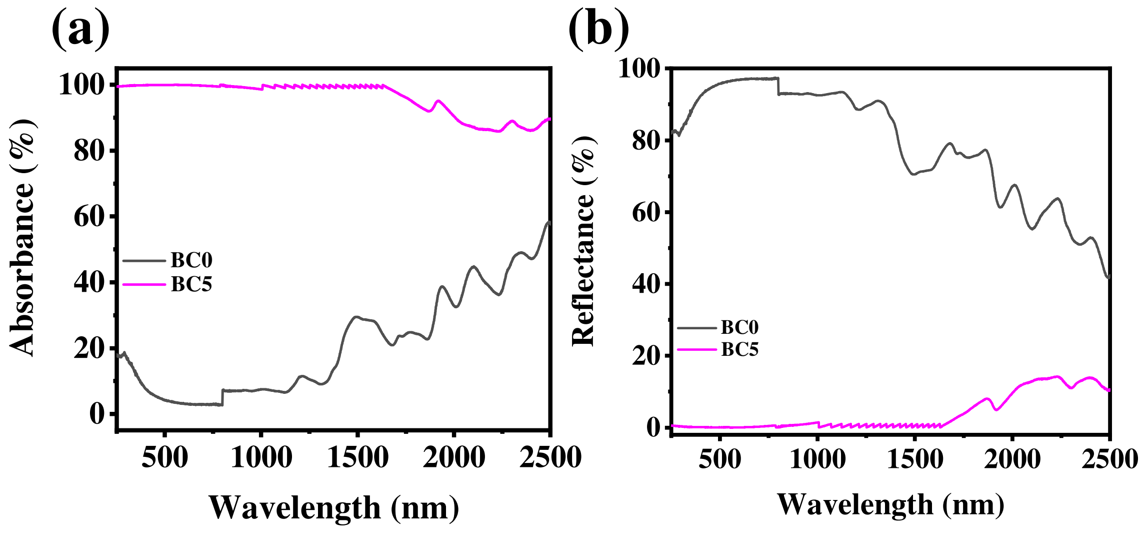

Figure S14. (a) Absorbance and (b) Reflectance spectra of samples determined by UV-vis.

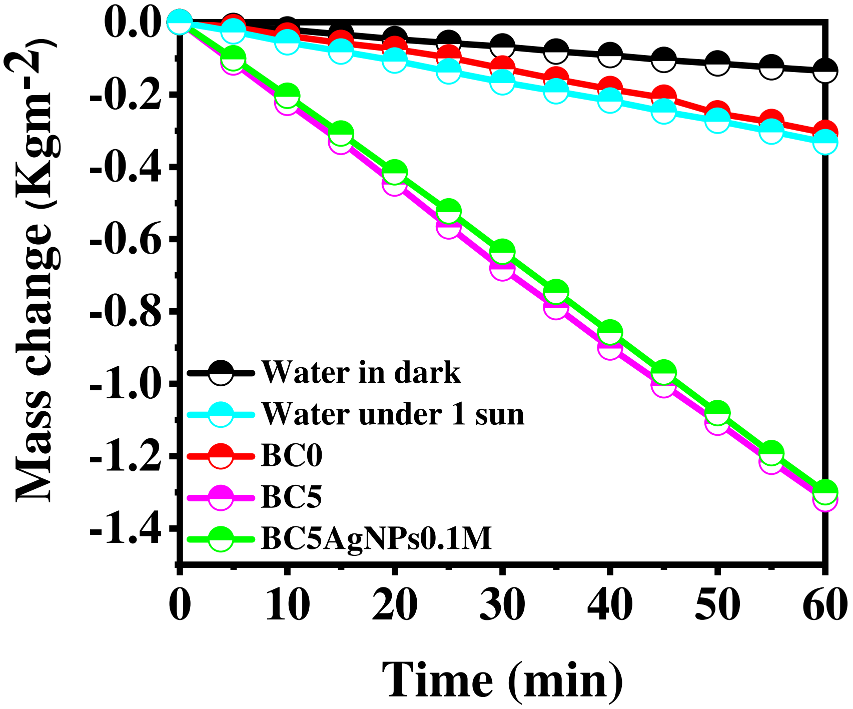

Figure S15. Mass change of water in dark and under 1 Sun irradiation of the cryogels.

Table S5. Comparison of solar-to-vapour conversion efficiencies of polyvinyl alcohol PVA based materials.

Sample name	***Conversion efficiency (%)***	***Reference***
PVA/Ni aerogel	78.3	[22]
PVA/nanocellulose scaffold	88	[23]
Wood-based PVA material	82.2	[24]
Molybdenum carbide–based polyvinyl alcohol hydrogel (MoCxPH)	83.6	[25]
PVA sponge	73.3	[26]
PVA synthetic tree	75	[27]
PVA based hybrid hydrogel	82.2	[28]
PVA based hydrogel	85.2	[29]
PVA based aerogel	79.3	[30]
PVA/CS/CuO aerogel	87.1	[31]
PVA sponge	73	[32]
PVA based aerogel	86	[2]
Fe_3O_4/PVA evaporator	73	[33]
**Biocomposite cryogel**	**81.9**	**This work**

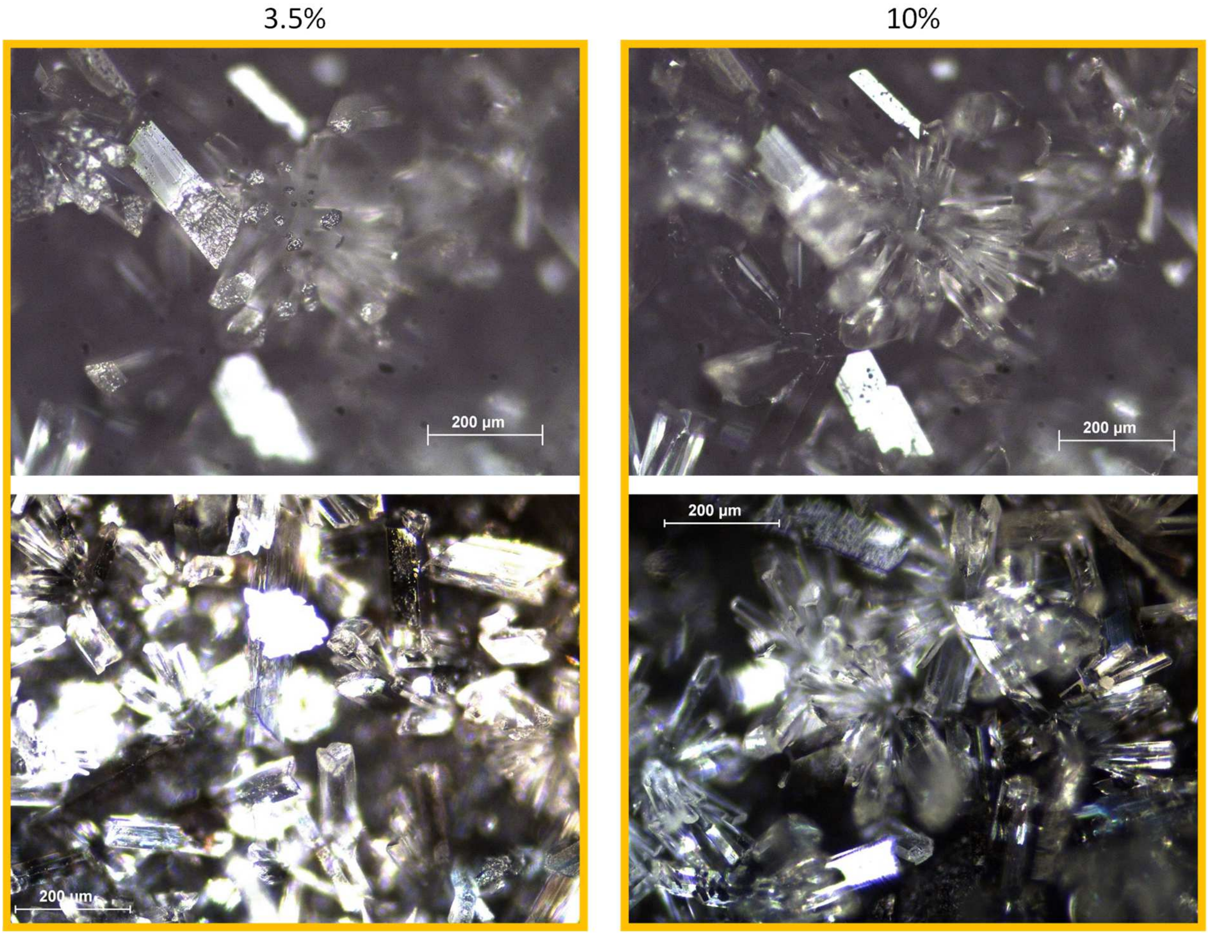

Figure S16. Images of salt crystals present on the BC5 sample surface taken by optical microscope.

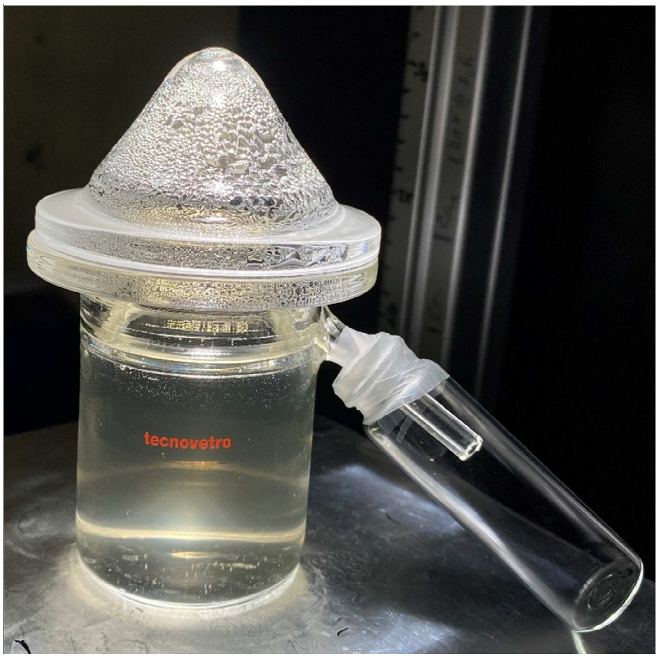

Figure S17. Glass chamber used for the process.

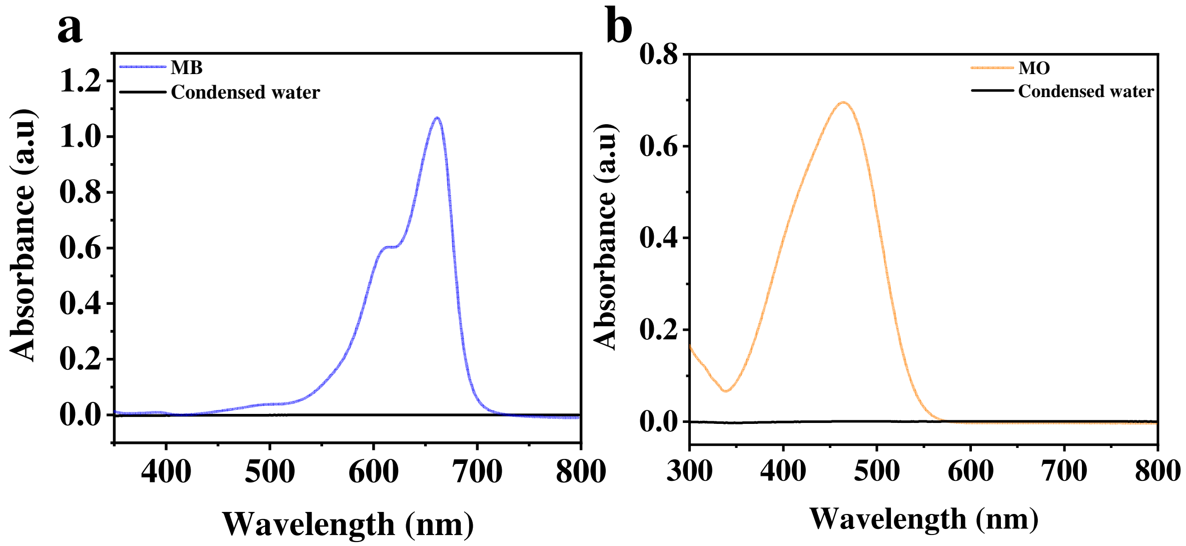

Figure S18. UV-vis absorption spectra of (a) MB and (b) MO aqueous solutions before and after solar purification.

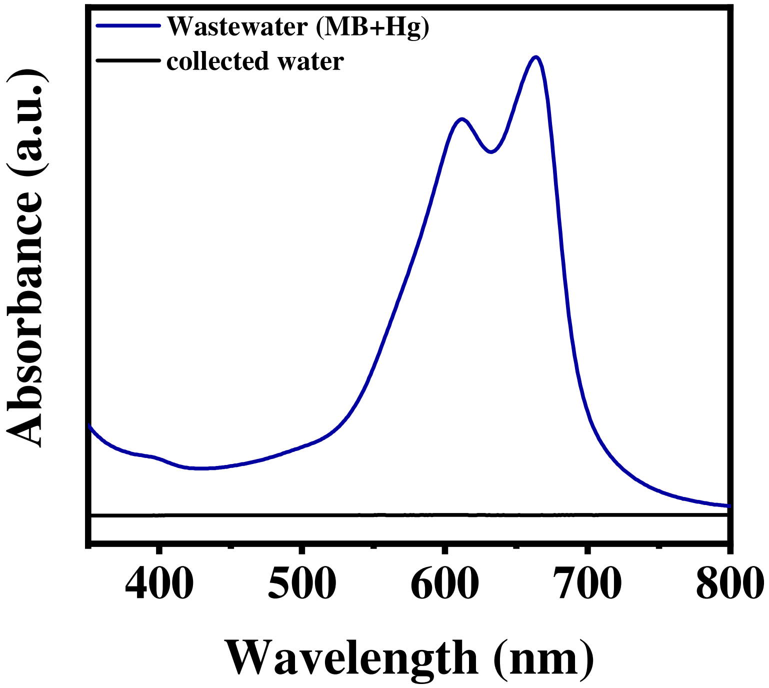

Figure S19. (a) UV-vis absorption spectrum of wastewater and purified water.

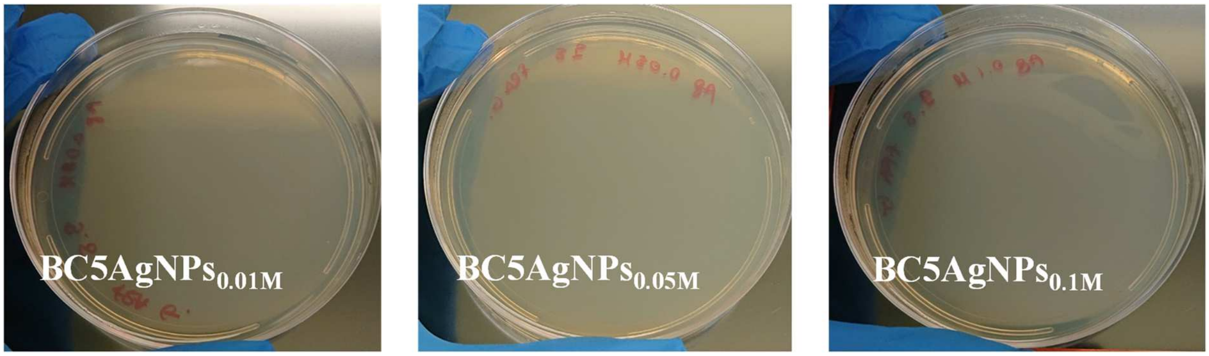

Figure S20. Representative photographs showing the bacterial (*Escherichia coli)* growth after liquid culture in contact with the different samples. The results demonstrate complete growth inhibition for all the tested samples after overnight treatment in solid culture, highlighting their strong antibacterial effect.

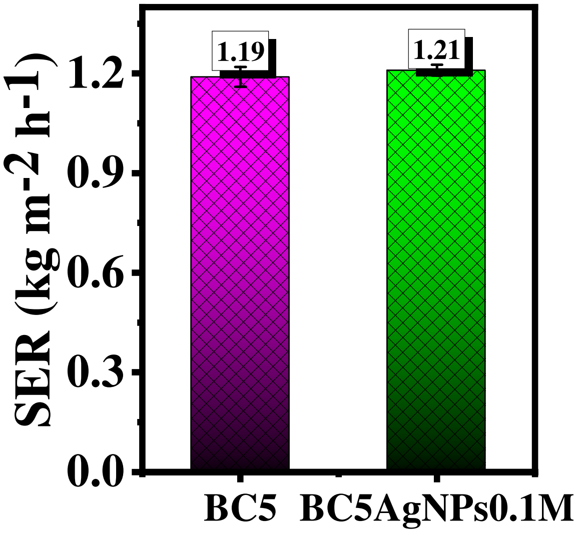

Figure S21. SER of BC5 and BC5AgNPs$_{0.1M}$ when both samples were floating separately on bacterial solution under 1 Sun irradiation.

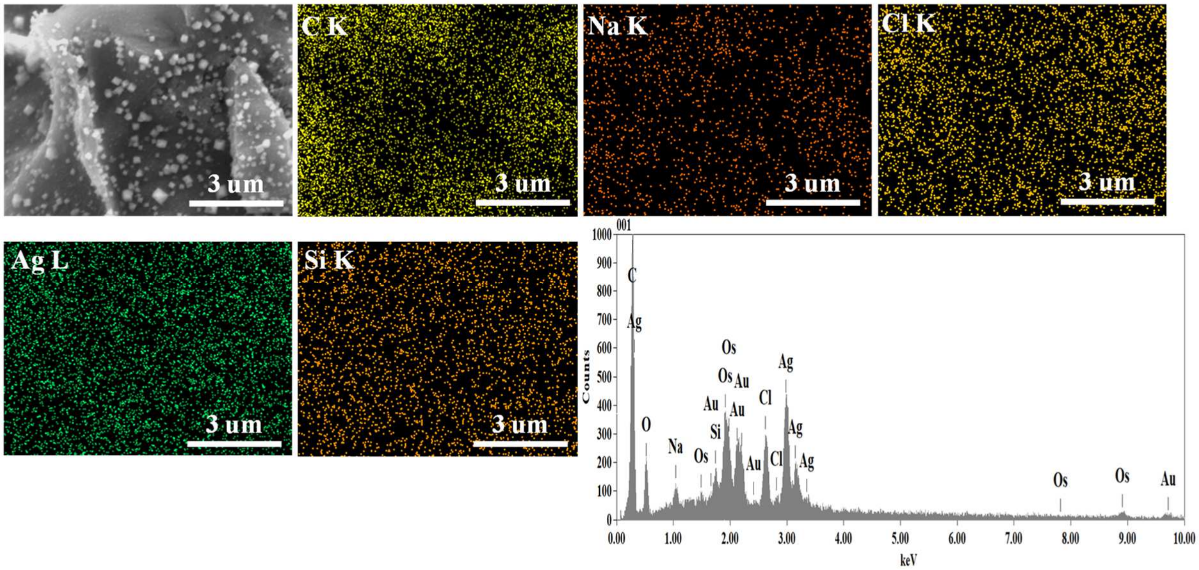

Figure S22. EDX of the AgNPs coated sample, when complete bacterial disruption was occurred.

Section S1. Removal of pollutants in static adsorption conditions

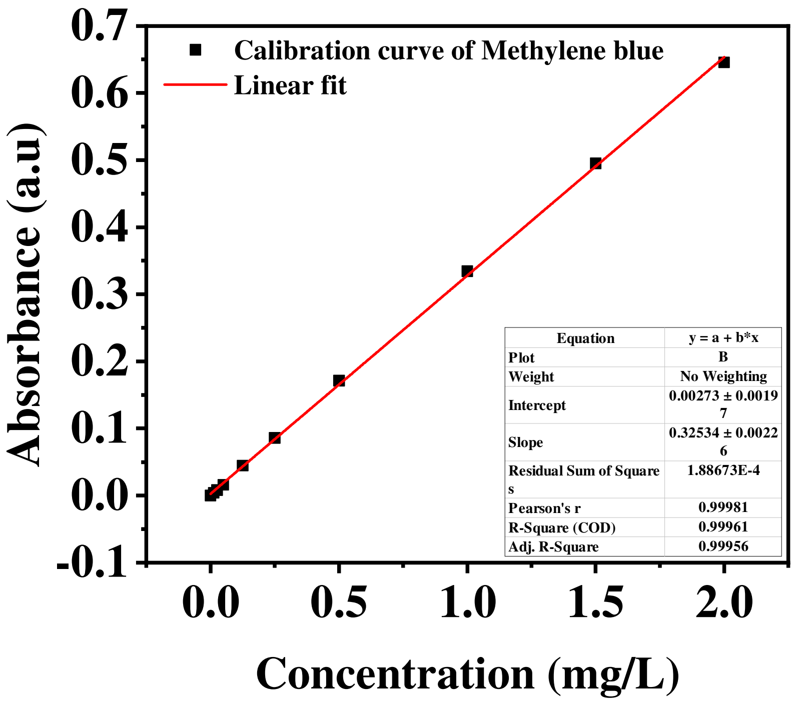

Figure S23. Calibration curve for the MB.

The adsorption capacity (Q_t) of the adsorbent material at equilibrium (Q_e) was calculated by the following equation:

$$Qt = \frac{Ci - Ct}{m} \times V \qquad \text{Equation S1}$$

C_i and C_t are the initial concentration and the concentration at time (t) of the MB solution (mg/L) respectively, m (g) is the mass of the adsorbent, and V (L) is the volume of MB solution. The concentration of the MB in the solutions was determined by first recording their absorption spectra in a range of 300-800 nm. Then the intensity of the peak at 664 nm was defined and the concentration of MB was calculated as follows:

$$C = (I_{664} - 0.00273) / 0.325 \qquad \text{Equation S2}$$

Note that 0.325 in Equation S2 is the slope value of a calibration curve resulting from the linear fitting of the I_{664} obtained by the absorption spectra of MB solutions of known concentrations (0 to 5 mg/ L) versus these concentrations (**Figure S23**). The linear equation was y =0.32534 × x+ 0.00273 with (R^2 = 0.99961), where y is I_{664} and x is the concentration.

As shown at the adsorption kinetics of MB (C_i=10 mgL^{-1}) on the BC5 **(Figure S24a)**, the MB adsorption capacity sharply increases within the first 1h then reaches the equilibrium after 4h interaction.

Figure S24b shows the evolution of the Q_e at MB solutions of different initial concentration. Q_e increases by increasing the initial MB concentration until the threshold of maximum adsorption capacity (Qmax) of 33.77 mg/g is reached.

Additionally, adsorption ability of cryogel was further conducted by dipping the BC5 in the Hg solution (10 ppm) for 24h in static condition. It is found out that the efficiency of Hg removal using BC5 was >90% (**Figure S24c**).

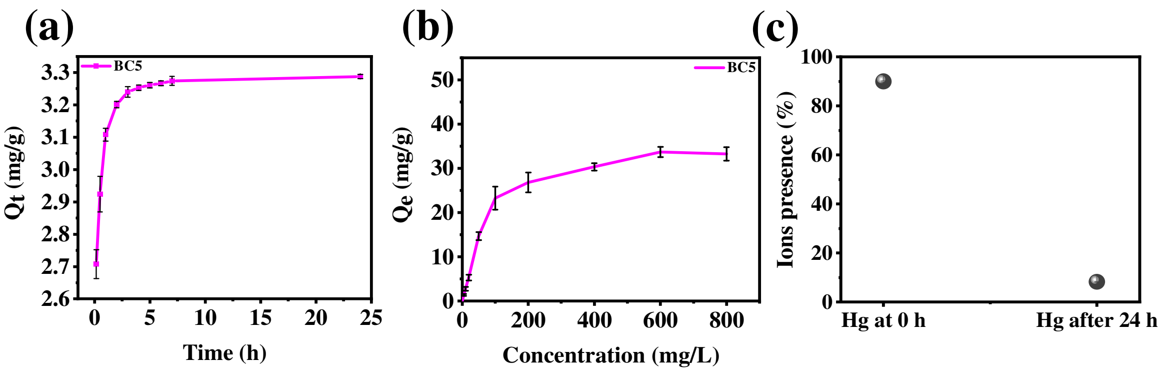

Figure S24. (a) Effect of contact time on the adsorption capacity for MB (Ci= 10 mgL^{-1}) (b) Effect of initial MB concentration on adsorption capacity after 24 h in contact with BC5 (c) Removal of Hg (10 ppm), when BC5 was dipped into its solution for 24h in static condition.

References

(1) Mu, P.; Bai, W.; Zhang, Z.; He, J.; Sun, H.; Zhu, Z.; Liang, W.; Li, A. Robust Aerogels Based on Conjugated Microporous Polymer Nanotubes with Exceptional Mechanical Strength for Efficient Solar Steam Generation. *J. Mater. Chem. A* **2018**, *6* (37), 18183–18190. https://doi.org/10.1039/C8TA05698F.

(2) Mu, P.; Song, L.; Geng, L.; Li, J. Aligned Attapulgite-Based Aerogels with Excellent Mechanical Property for the Highly Efficient Solar Steam Generation. *Sep. Purif. Technol.* **2021**, *271*, 118869. https://doi.org/https://doi.org/10.1016/j.seppur.2021.118869.

(3) Mu, P.; Bai, W.; Fan, Y.; Zhang, Z.; Sun, H.; Zhu, Z.; Liang, W.; Li, A. Conductive Hollow Kapok Fiber-PPy Monolithic Aerogels with Excellent Mechanical Robustness for Efficient Solar Steam Generation. *J. Mater. Chem. A* **2019**, *7* (16), 9673–9679. https://doi.org/10.1039/C8TA12243A.

(4) Song, L.; Geng, L.; Tian, Y.; Mu, P.; Li, J. Robust Superhydrophilic Attapulgite-Based Aligned Aerogels for Highly Efficient and Stable Solar Steam Generation in Harsh Environments. *J. Mater. Chem. A* **2021**, *9* (40), 23117–23126. https://doi.org/10.1039/D1TA07042H.

(5) Wang, F.; Wei, D.; Li, Y.; Chen, T.; Mu, P.; Sun, H.; Zhu, Z.; Liang, W.; Li, A. Chitosan/Reduced Graphene Oxide-Modified Spacer Fabric as a Salt-Resistant Solar Absorber for Efficient Solar Steam Generation. *J. Mater. Chem. A* **2019**, *7* (31), 18311–18317. https://doi.org/10.1039/C9TA05859A.

(6) Wang, S.; Niu, Y.; Ye, X.; Liu, F.; Su, M.; Zhu, Z.; Sun, H.; Li, J.; Liang, W.; Li, A. Robustly Inorganic Solar Steam Generator Derived from Hollow Glass Microspheres

Based Composites for Desalination. *Sol. RRL* **2021**, *5* (12), 2100771. https://doi.org/https://doi.org/10.1002/solr.202100771.

(7) Lin, X.; Wang, P.; Hong, R.; Zhu, X.; Liu, Y.; Pan, X.; Qiu, X.; Qin, Y. Fully Lignocellulosic Biomass-Based Double-Layered Porous Hydrogel for Efficient Solar Steam Generation. *Adv. Funct. Mater.* **2022**, *32* (51), 2209262. https://doi.org/https://doi.org/10.1002/adfm.202209262.

(8) Ni, A.; Lin, P.; Wang, X.; Fu, D.; Hua, S.; Pei, D.; Li, S.; Han, X.; Xia, Y.; Zhang, T. Facile Preparation of High Strength Aerogel Evaporator for Efficient Solar-Driven Water Purification. *Sustain. Mater. Technol.* **2022**, *32*, e00443. https://doi.org/https://doi.org/10.1016/j.susmat.2022.e00443.

(9) Irshad, M. S.; Wang, X.; Abbasi, M. S.; Arshad, N.; Chen, Z.; Guo, Z.; Yu, L.; Qian, J.; You, J.; Mei, T. Semiconductive, Flexible MnO2 NWs/Chitosan Hydrogels for Efficient Solar Steam Generation. *ACS Sustain. Chem. Eng.* **2021**, *9* (10), 3887–3900. https://doi.org/10.1021/acssuschemeng.0c08981.

(10) Liu, F.; Liang, W.; Wang, C.; Xiao, C.; He, J.; Zhao, G.; Zhu, Z.; Sun, H.; Li, A. Superhydrophilic and Mechanically Robust Phenolic Resin as Double Layered Photothermal Materials for Efficient Solar Steam Generation. *Mater. Today Energy* **2020**, *16*, 100375. https://doi.org/https://doi.org/10.1016/j.mtener.2019.100375.

(11) Li, Q.; Zhao, X.; Li, L.; Hu, T.; Yang, Y.; Zhang, J. Facile Preparation of Polydimethylsiloxane/Carbon Nanotubes Modified Melamine Solar Evaporators for Efficient Steam Generation and Desalination. *J. Colloid Interface Sci.* **2021**, *584*, 602–609. https://doi.org/https://doi.org/10.1016/j.jcis.2020.10.002.

(12) Xiao, C.; Chen, L.; Mu, P.; Jia, J.; Sun, H.; Zhu, Z.; Liang, W.; Li, A. Sugarcane-Based Photothermal Materials for Efficient Solar Steam Generation. *ChemistrySelect* **2019**, *4* (27), 7891–7895. https://doi.org/https://doi.org/10.1002/slct.201901889.

(13) Zhao, F.; Zhou, X.; Shi, Y.; Qian, X.; Alexander, M.; Zhao, X.; Mendez, S.; Yang, R.; Qu, L.; Yu, G. Highly Efficient Solar Vapour Generation via Hierarchically Nanostructured Gels. *Nat. Nanotechnol.* **2018**, *13* (6), 489–495. https://doi.org/10.1038/s41565-018-0097-z.

(14) Gong, J. P.; Katsuyama, Y.; Kurokawa, T.; Osada, Y. Double-Network Hydrogels with Extremely High Mechanical Strength. *Adv. Mater.* **2003**, *15* (14), 1155–1158. https://doi.org/https://doi.org/10.1002/adma.200304907.

(15) Jia, C.; Li, Y.; Yang, Z.; Chen, G.; Yao, Y.; Jiang, F.; Kuang, Y.; Pastel, G.; Xie, H.; Yang, B.; Das, S.; Hu, L. Rich Mesostructures Derived from Natural Woods for Solar Steam Generation. *Joule* **2017**, *1* (3), 588–599. https://doi.org/https://doi.org/10.1016/j.joule.2017.09.011.

(16) Chen, L.; Ding, Y.; Gong, J.; Xie, H.; Qu, J.; Niu, R. Molecular Engineering of Biomass-Derived Hybrid Hydrogels for Solar Water Purification. *J. Colloid Interface Sci.* **2022**, *626*, 231–240. https://doi.org/https://doi.org/10.1016/j.jcis.2022.06.145.

(17) Liu, F.; Zhao, B.; Wu, W.; Yang, H.; Ning, Y.; Lai, Y.; Bradley, R. Low Cost, Robust, Environmentally Friendly Geopolymer–Mesoporous Carbon Composites for Efficient Solar Powered Steam Generation. *Adv. Funct. Mater.* **2018**, *28* (47), 1803266. https://doi.org/https://doi.org/10.1002/adfm.201803266.

(18) Xie, X.; Liu, L.; Zhang, L.; Lu, A. Strong Cellulose Hydrogel as Underwater

Superoleophobic Coating for Efficient Oil/Water Separation. *Carbohydr. Polym.* **2020**, *229* (July 2019), 115467. https://doi.org/10.1016/j.carbpol.2019.115467.

(19) Chen, J.; Zuo, K.; Li, B.; Hu, J.; Liu, W.; Xia, D.; Lin, L.; Liang, J.; Li, X. Fungal Hypha-Derived Freestanding Porous Carbon Pad as a High-Capacity Electrode for Water Desalination in Membrane Capacitive Deionization. *Chem. Eng. J.* **2022**, *433*, 133781. https://doi.org/https://doi.org/10.1016/j.cej.2021.133781.

(20) Maleki, M.; Arabpour Roghabadi, F.; Sadrameli, S. M. High-Performance Solar Steam Generator Using Low-Cost Biomass Waste Photothermal Material and Engineering of the Structure. *ACS Omega* **2022**, *7* (44), 39895–39906. https://doi.org/10.1021/acsomega.2c04146.

(21) Luo, J.; Fan, C.; Zhou, X. Functionalized Graphene Oxide/Carboxymethyl Chitosan Composite Aerogels with Strong Compressive Strength for Water Purification. *J. Appl. Polym. Sci.* **2021**, *138* (12), 50065. https://doi.org/https://doi.org/10.1002/app.50065.

(22) Kim, C.; Ryu, Y.; Shin, D.; Urbas, A. M.; Kim, K. Efficient Solar Steam Generation by Using Metal-Versatile Hierarchical Nanostructures for Nickel and Gold with Aerogel Insulator. *Appl. Surf. Sci.* **2020**, *517*, 146177. https://doi.org/https://doi.org/10.1016/j.apsusc.2020.146177.

(23) Yang, L.; Li, N.; Guo, C.; He, J.; Wang, S.; Qiao, L.; Li, F.; Yu, L.; Wang, M.; Xu, X. Marine Biomass-Derived Composite Aerogels for Efficient and Durable Solar-Driven Interfacial Evaporation and Desalination. *Chem. Eng. J.* **2021**, *417*, 128051. https://doi.org/https://doi.org/10.1016/j.cej.2020.128051.

(24) Lu, Y.; Dai, T.; Fan, D.; Min, H.; Ding, S.; Yang, X. Turning Trash into Treasure: Pencil

Waste–Derived Materials for Solar-Powered Water Evaporation. *Energy Technol.* **2020**, *8* (10), 2000567. https://doi.org/https://doi.org/10.1002/ente.202000567.

(25) Yu, F.; Ming, X.; Xu, Y.; Chen, Z.; Meng, D.; Cheng, H.; Shi, Z.; Shen, P.; Wang, X. Quasimetallic Molybdenum Carbide–Based Flexible Polyvinyl Alcohol Hydrogels for Enhancing Solar Water Evaporation. *Adv. Mater. Interfaces* **2019**, *6* (24), 1901168. https://doi.org/https://doi.org/10.1002/admi.201901168.

(26) Zhang, Z.; Mu, P.; He, J.; Zhu, Z.; Sun, H.; Wei, H.; Liang, W.; Li, A. Facile and Scalable Fabrication of Surface-Modified Sponge for Efficient Solar Steam Generation. *ChemSusChem* **2019**, *12* (2), 426–433. https://doi.org/https://doi.org/10.1002/cssc.201802406.

(27) Shao, Y.; Tang, J.; Li, N.; Sun, T.; Yang, L.; Chen, D.; Zhi, H.; Wang, D.; Liu, H.; Xue, G. Designing a Bioinspired Synthetic Tree by Unidirectional Freezing for Simultaneous Solar Steam Generation and Salt Collection. *EcoMat* **2020**, *2* (1), e12018. https://doi.org/https://doi.org/10.1002/eom2.12018.

(28) Tan, M.; Wang, J.; Song, W.; Fang, J.; Zhang, X. Self-Floating Hybrid Hydrogels Assembled with Conducting Polymer Hollow Spheres and Silica Aerogel Microparticles for Solar Steam Generation. *J. Mater. Chem. A* **2019**, *7* (3), 1244–1251. https://doi.org/10.1039/C8TA10057H.

(29) Li, J.; Yan, L.; Li, X.; Song, W.; Li, Y. Porous Polyvinyl Alcohol/Biochar Hydrogel Induced High Yield Solar Steam Generation and Sustainable Desalination. *J. Environ. Chem. Eng.* **2022**, *10* (3), 107690. https://doi.org/https://doi.org/10.1016/j.jece.2022.107690.

(30) Huang, Z.; Li, S.; Cui, X.; Wan, Y.; Xiao, Y.; Tian, S.; Wang, H.; Li, X.; Zhao, Q.; Lee, C.-S. A Broadband Aggregation-Independent Plasmonic Absorber for Highly Efficient Solar Steam Generation. *J. Mater. Chem. A* **2020**, *8* (21), 10742–10746. https://doi.org/10.1039/D0TA01980A.

(31) Tian, Y.; Wang, X.; Gu, Y.; Mu, X.; Wang, P.; Wei, A.; Zhang, J.; Chen, Y.; Sun, Z.; Jia, L.; Zhao, Z.; Zhou, J.; Miao, L. Versatile PVA/CS/CuO Aerogel with Superior Hydrophilic and Mechanical Properties towards Efficient Solar Steam Generation. *Nano Sel.* **2021**, *2* (12), 2380–2389. https://doi.org/https://doi.org/10.1002/nano.202100125.

(32) Gao, X.; Lan, H.; Li, S.; Lu, X.; Zeng, M.; Gao, X.; Wang, Q.; Zhou, G.; Liu, J.-M.; Naughton, M. J.; Kempa, K.; Gao, J. Artificial Mushroom Sponge Structure for Highly Efficient and Inexpensive Cold-Water Steam Generation. *Glob. Challenges* **2018**, *2* (12), 1800035. https://doi.org/https://doi.org/10.1002/gch2.201800035.

(33) Song, L.; Zhang, X.-F.; Wang, Z.; Zheng, T.; Yao, J. Fe3O4/Polyvinyl Alcohol Decorated Delignified Wood Evaporator for Continuous Solar Steam Generation. *Desalination* **2021**, *507*, 115024. https://doi.org/https://doi.org/10.1016/j.desal.2021.115024.
